# Tumor-secreted lactate contributes to an immunosuppressive microenvironment and affects CD8 T-cell infiltration in glioblastoma

**DOI:** 10.3389/fimmu.2023.894853

**Published:** 2023-04-12

**Authors:** Zeyu Wang, Ziyu Dai, Hao Zhang, Xisong Liang, Xun Zhang, Zhipeng Wen, Peng Luo, Jian Zhang, Zaoqu Liu, Mingyu Zhang, Quan Cheng

**Affiliations:** ^1^ Department of Neurosurgery, Xiangya Hospital, Central South University, Changsha, Hunan, China; ^2^ National Clinical Research Center for Geriatric Disorders, Xiangya Hospital, Central South University, Changsha, Hunan, China; ^3^ MRC Centre for Regenerative Medicine, Institute for Regeneration and Repair, University of Edinburgh, Edinburgh, United Kingdom; ^4^ Department of Pharmacy, The Affiliated Hospital of Guizhou Medical University, Guizhou Medical University, Guiyang, Guizhou, China; ^5^ Department of Oncology, Zhujiang Hospital, Southern Medical University, Guangzhou, China; ^6^ Department of Interventional Radiology, The First Affiliated Hospital of Zhengzhou University, Zhengzhou, Henan, China; ^7^ Clinical Diagnosis and Therapy Center for Gliomas of Xiangya Hospital, Central South University, Changsha, Hunan, China; ^8^ Department of Clinical Pharmacology, Xiangya Hospital, Central South University, Changsha, Hunan, China

**Keywords:** glioblastoma, CD8+ T cells, interferon-gamma, immunocytes, chemotherapy

## Abstract

**Introduction:**

Glioblastoma is a malignant brain tumor with poor prognosis. Lactate is the main product of tumor cells, and its secretion may relate to immunocytes’ activation. However, its role in glioblastoma is poorly understood.

**Methods:**

This work performed bulk RNA-seq analysis and single cell RNA-seq analysis to explore the role of lactate in glioblastoma progression. Over 1400 glioblastoma samples were grouped into different clusters according to their expression and the results were validated with our own data, the xiangya cohort. Immunocytes infiltration analysis, immunogram and the map of immune checkpoint genes’ expression were applied to analyze the potential connection between the lactate level with tumor immune microenvironment. Furthermore, machine learning algorithms and cell-cell interaction algorithm were introduced to reveal the connection of tumor cells with immunocytes. By co-culturing CD8 T cells with tumor cells, and performing immunohistochemistry on Xiangya cohort samples further validated results from previous analysis.

**Discussion:**

In this work, lactate is proved that contributes to glioblastoma immune suppressive microenvironment. High level of lactate in tumor microenvironment can affect CD8 T cells’ migration and infiltration ratio in glioblastoma. To step further, potential compounds that targets to samples from different groups were also predicted for future exploration.

## Introduction

Glioblastoma (GBM) is a malignant tumor derived from the central nervous system, with median survival time <15 months (1). GBM manifests a high aggressive growth pattern, high recurrence ratio, and high resistance to chemo- and radiotherapy, which together contribute to its poor prognosis (2). Targeted therapies, such as RTK inhibitors, have not proven successful and are likely undermined by genetic heterogeneity (3, 4). Apart from maximal surgical removal, current clinical strategies including chemotherapy (temozolomide) and radiotherapy can improve patients’ survival outcome but are never curative. Recent research reported that GBM can be grouped into different subtypes based on genetic characteristic, and those subtypes showed different responses to treatment (5–7). Therefore, exploring the genetic characteristics of GBM may assist in understanding tumorigenesis, tumor recurrence, and innovating novel treatments.

The Warburg effect was first proposed in 1924 and described the metabolism rewiring in tumor cells, and this effect has been proved to modulate tumor progression (8) and the tumor-immunosuppressive microenvironment (9). Moreover, the product of the Warburg effect, lactate, was also reported as a mediator between tumor cells and other cells, like macrophage polarization (10), cytotoxic T cells (11), CD4 T cells (12), fibroblasts (13), dendritic cells (14), regulatory T cells (15), and natural killer cells (16). Therefore, lactate plays a critical role in bridging tumor cells and other cells. In GBM, accumulation of lactate was believed to be connected with GBM invasiveness (17), to modulate tumor cell–epithelial cell crosstalk (18), and to be associated with tumor resistance to chemotherapy (19). Nevertheless, the role of lactate in GBM remains elusive.

The GBM immunosuppressive microenvironment has gained a lot of attention in recent years since GBM, unlike other tumors like melanoma, resistant to immunotherapy (20–22). Several studies reported that T cells, peripherally derived macrophages, dendritic cells, microglia, and myeloid cells contributed to the GBM immunosuppressive microenvironment and impaired the efficiency of immunotherapy (23–26). Meanwhile, INFG has been reported to be involved in various tumors’ immunosuppressive microenvironment including hepatocellular carcinoma (27), neuroblastoma (28), and breast cancer (29). Interestingly, research which obtained mesenchymal GBM by genetic manipulation of neural stem cells proposed that GBM immune evasion and its immunosuppressive microenvironment were closely related to GBM subtypes (1). Therefore, the influence of GBM cells on immunocytes may contribute to its immunosuppressive microenvironment, which together further interfere with GBM response to immunotherapy. However, the mechanism on how GBM cells affect immunocytes is poorly investigated.

In this work, we collected lactate-associated genes (LAGs) and explored their role in GBM. A consensus cluster analysis was performed based on LAGs to classify 1,410 GBM samples, the GBM meta cohort (30, 31), into two clusters. Cluster 1 samples showed worse prognosis than cluster 2 samples and produced more lactate than cluster 2 samples based on LAG expression. Biofunction prediction based on bulk RNA-seq analysis and single-cell RNA-seq analysis suggested that immunocytes’ function in cluster 1 was affected. Moreover, cell–cell interaction and immunogram (32) revealed that GBM cells with high lactate accumulation may modulate immunocytes (including microglia, CD8 T cells, and peripheral blood macrophage) and tumor response to interform gamma (IFNG), which together result in an immunosuppressive microenvironment in GBM. An *in vitro* experiment supported that CD8 T-cell infiltration and migration were influenced by lactate accumulation. In the end, sensitive compounds based on the cluster model were predicted, which may improve GBM patients’ prognosis. Therefore, lactate may modulate the GBM immunosuppressive microenvironment through targeting immunocytes and modulate tumor response to IFNG.

## Materials and methods

### Data processing

The GBM meta cohort was set as the training dataset, whereas TCGA GBM array dataset and the Xiangya GBM cohort were treated as validation datasets. The GBM meta cohort contained 1,410 GBM samples from CGGA_325, CGGA_693, GSE108474, GSE42669, GSE4271, GSE43378, GSE4412, GSE74187, GSE7696, GSE83300, and TCGA data. The definition of GBM samples is from corresponding database. R package “sva” was used to eliminate the batch effect during data combination. More details can be found in a previous work (31).

Data on TCGA GBM array cohort containing 539 GBM samples were downloaded from UCSC Xena (https://xenabrowser.net/) (33). Data on the Xiangya cohort containing 73 GBM samples were processed as previously described (31) and were (ID: HRA001618) uploaded onto the China National Center for Bioinformation (https://www.cncb.ac.cn/). Expression data of glioma cell lines were downloaded from the Cancer Cell Line Encyclopedia (CCLE, https://sites.broadinstitute.org/ccle). All high-throughput sequencing datasets were transformed into log2(TPM+1) for subsequent analysis.

Raw data of GBM samples (ID: GSE138794, GSE84465, SCP50, GSE131928) were downloaded and combined into one cohort for single-cell RNA-seq analysis. Datasets were combined with R package “Seurat.” Meanwhile, R package “NormalizeData” was used for data procession, “scCATCH” was used for cell annotation, “infercnv” was used for tumor cell identification, and “TSNE” was introduced for visualization.

### Consensus cluster analysis

Samples were subdivided into different groups by introducing R package “Consensus Cluster Plus” (34). The optimum group number was decided by introducing the cumulative distribution function plots and consensus matrices.

### Epigenetic alternation analysis

Single-nucleotide polymorphisms (SNPs) and somatic copy number variations (CNVs) based on TCGA GBM array were analyzed with R package “maftools.” Associated SNPs and CNV data were downloaded from UCSC Xena. GISTIC analysis was introduced, and variation peaks were generated using GISTIC 2.0 analysis (https://gatk.broadinstitute.org). R packages “ggplot2,” “ggsignif,” and “gg.gap” were used for visualization.

### Enrichment analysis based on GSVA and GSEA

GO pathway-related gene lists were downloaded from http://www.gsea-msigdb.org/gsea/msigdb. The GSVA score of those pathways was calculated with R package “GSVA.” GSEA enrichment was performed with R package “clusterProfiler.”

### Machine learning algorithms

A support vector machine (R package ‘e1071’) was used to learn the characteristics of the cluster model, and this model was reproduced on glioma cell lines. Another two machine learning methods, Boruta and XGboost (R packages ‘Boruta’ and “xgboost”), were introduced to identify the immunocyte characteristics between cluster 1 and cluster 2. The interaction of the immunocyte-characteristic and immunocyte infiltration ratio is shown in a Venn diagram (R package “VennDiagram”).

### Immunocyte infiltration and immune escape-associated genes

CIBERSORT and xCell algorithm were performed for immunocyte infiltration ratio analysis with R package “CIBERSORT” (35, 36) and “xCell” (37), respectively.

An immunogram was introduced to illustrate the immune-associated pathway activation difference based on the cluster model. A gene list was obtained from a previous work (32), and R packages “ssGSEA” and “ggradar” were introduced for visualization.

Immune escape-associated genes were collected from a previous work (38) and visualized with R package “ggplot2.”

### Cell–cell interaction

R package “CellChat” was used to predict the interaction between tumor cells and other cells in the tumor immune landscape based on ligand–receptor pairs (39).

### Cell culture

Three GBM cell lines (U251MG, U87MG, and A172) were purchased from BeNa Culture Collection (https://www.bncc.org.cn/). CD8 T cells were isolated from donor. Individuals’ informed consent were signed and verified by the ethics committee. Cells were cultured in high-glucose DMEM with 10% fetal bovine serum at 37°C and 5% CO_2_.

For CD8 T-cell isolation, lymphocyte separation medium was added to blood. A medium white layer was extracted gently after centrifuging at 2,000 rpm for 20 min. Platelets were removed by centrifuging at 1,500 rpm for 10 min. Red blood cells lysis buffer was added to lyse red blood cells for 30 min. Finally, CD8 MicroBeads were added for CD8 T-cell isolation.

### Lactate assay

2 × 10^5^ U251MG, U87MG, and A172 cells were seeded into a six-well plate. Cultural medium was collected after culturing for 48 h, and a lactate concentration kit (Nanjing Jiancheng Bioengineering Institute, China) was used for calculating lactate concentration.

### Migration assay

A 5-µm Transwell chamber was used to coculture GBM cell lines and CD8 T cells. 3 × 10^5^ U87MG/A172 cells were seeded into the lower chamber, and 3 × 10^5^ CD8 T cells were cultured in the upper chamber. After coculturing CD8+ T cells and tumor cells for 48 h, cells in the lower chamber were harvested and calculated by flow cytometry. For the external lactate group, 7.5 mmol/l lactate medium was added to the lower chamber.

### Immunohistochemistry

Xiangya GBM samples from different clusters were selected for immunohistochemistry (IHC) as previously described (40). Glioma tissues were collected and written informed consent was obtained from patients. The included glioma tissues were approved by the Ethics Committee of Xiangya Hospital, Central South University. In brief, slides were incubated with anti-CD8 antibody (66868-1-Ig, Proteintech) at 4°C overnight. Signals were visualized under standard protocols, and images were captured by introducing an Olympus inverted microscope.

### Compound prediction

Drug sensitivity prediction based on PRISM, CTRP, and CellMiner databases was conducted as described in previous works (41, 42). Briefly, compound sensitivity was evaluated as an AUC score; a lower AUC score indicates higher sensitivity. The sensitivity of samples from the GBM meta cohort was predicted with R package “pRRophetic.”

### Statistical analysis

A normality test was performed first to determine data distribution. Then, Student’s t test or ANOVA was used for normally distributed data; Wilcoxon test and Kruskal–Wallis test were introduced for non-normally distributed data to examine the difference between two or multiple groups, respectively. Kaplan–Meier curves with the log-rank test were generated to illustrate patients’ prognosis difference. The Wilcoxon rank-sum test was used to compare the difference between various groups. Results from a pan-cancer analysis were analyzed using GSCALite (http://bioinfo.life.hust.edu.cn/web/GSCALite/) (43). Results from the bulk RNA-seq analysis and single-cell RNA-seq analysis were analyzed with R (version 4.1.2).

## Results

### Lactate-associated genes are dysregulated in various tumor types

To illustrate the role of lactate level in tumor, we first collected key LAGs from GO gensets (GO_LACTATE_METABOLIC_PROCESS; GO_LACTATE_TRANSPORT), including HIF1A, LDHA, LDHB, PFKFB2, SLC16A1, SLC16A3, SLC16A7, SLC16A8, and UEVLD. HIF1A is a critical trigger of glycolysis as a response to hypoxia. LDHA and LDHB are lactate dehydrogenases regulating the transition between pyruvate and lactate. The SLC family is responsible for extracellular lactate transport. PFKFB2 regulates the generation of fructose-2,6-bisphosphate and controls glycolysis. UEVLD is believed to enable the activity of oxidoreductase, which is a critical enzyme that controls the glycolysis bypass pathway and pentose phosphate pathway (44).

**Figure 1:**
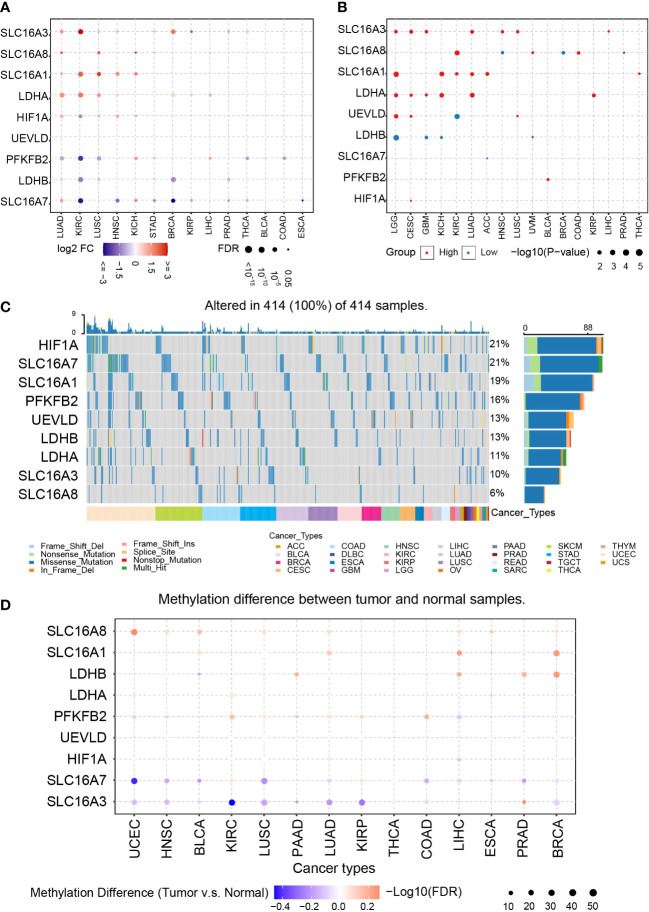
Pan-cancer analysis of LAGs. **(A)** The expression of LAGs in various tumors. **(B)** The survival analysis of LAGs in various tumors. **(C)** The SNPs of LAGs in various tumors. **(D)** The methylation status of LAGs in various tumors.

LAG expression was first mapped in various tumor types compared with their paired normal tissue in TCGA (**Figure 1A**). As illustrated, genes like SLCA6A3, SLC16A8, SLC16A1, and LDHA have a higher expression in LUAD and LUSC than paired normal tissues, whereas the expression of PFKFB2 decreases in those tumors. Then, survival analysis indicates that a high expression of SLCA6A3, SLC16A1, and LDHA and a low expression of LDHB are recognized as a tumor progression promotor in some tumors (**Figure 1B**). Therefore, dysregulated LAG expression is common in various tumor types and is highly connected to tumor prognosis.

Next, SNPs, methylation, and copy number variations (CNVs) are analyzed to explore their potential connections with dysregulated LAGs. Among all tumor types in TCGA, the most common mutated genes are HIF1A and SLC16A7, which accounts for 21% in all samples (**Figure 1C**). Over 10% samples carry mutated SLC16A1 (19%), PFKFB2 (16%), UEVLD (13%), LDHB (13%), and LDHA (11%), and the proportion of mutated SLC16A8 in all samples is 6%. Interestingly, genes that have a higher mutation ratio, like SLC16A7, PFKFB2, and HIF1A, are biomarkers that have no relationship with tumor prognosis.

The methylation difference between tumor and their paired normal samples was also investigated. LAGs like SLC16A8, SLC16A1, and LDHB have a higher methylation ratio in tumor tissue than in normal tissue; a lower methylation ratio of SLC16A7 and SLC16A3 is noticed in that comparison (**Figure 1D**). Among most of these tumor types, a negative correlation between gene methylation and mRNA expression was revealed by calculating the Spearman correlation, including HIF1A, SLC16A1, UEVLD, SLC16A8, PFKFB2, LDHB, and SLC16A3 (**Supplementary Figures 1A, B**). In contrast, a positive correlation tendency of SLC16A7 was found.

CNVs based on homozygous or heterozygous amplification and deletion were explored. Homozygous amplification of SLC16A3 and PFKFB2 was only found in BRCA, CHOL, LIHC, and OV (**Supplementary Figure 2A**). Homozygous deletion of LAGs was not identified. Moreover, massive heterozygous amplification and deletion of LAGs were located in various tumor types (**Supplementary Figure 2B**).

In summary, dysregulated LAGs are common in various tumor types and are associated with tumor progression. Previous studies highlighted that dysregulation of LAGs can affect tumor progression (45). For instance, LDHB promoter methylation is associated with breast cancer progression (46). HIF1A controls GBM growth and sensitivity to treatments through the PDGFD–PDGFRα axis (47). Therefore, dysregulated LAGs may affect GBM prognosis.

### The prognosis of glioblastoma patients in cluster 1 is worse than that in cluster 2

Considering that GBM is a malignant tumor and its relationship with LAGs is elusive, we focused on exploring its role in GBM. The GBM meta dataset was set as the training cohort, whereas TCGA GBM-array dataset and Xiangya dataset were used for validation. As for the validation cohort, there are 539 samples in TCGA GBM-array and 73 samples in the Xiangya cohort.

**Figure 2:**
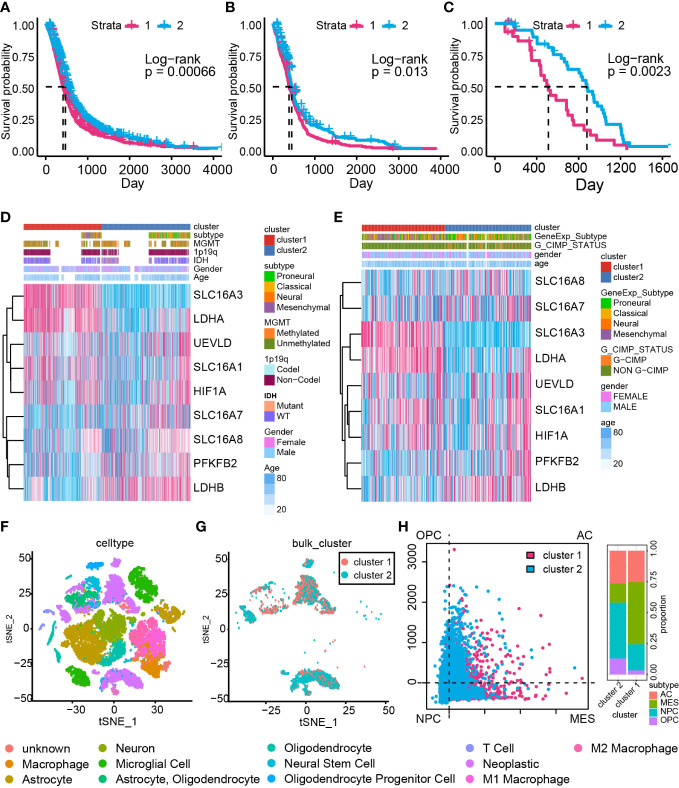
The cluster model based on LAGs. Overall survival analysis based on the GBM meta cohort **(A)**, TCGA GBM array cohort **(B)**, and Xiangya cohort **(C)**. The heatmap of LAGs in the GBM meta cohort **(D)** and TCGA GBM array cohort **(E)** along with clinical features. **(F)** Components of data from single-cell RNA-seq analysis. **(G)** The cluster model in data from single-cell RNA-seq analysis. **(H)** The percentage of MES-like, AC-like, OPC-like, and NPC-like cells in the cluster model.

The consensus cluster analysis classified samples in the GBM meta cohort into two groups, cluster 1 and cluster 2 (**Supplementary Figures 3A, B**). The support vector machine was used to identify the main difference characteristics between two groups and regroup samples in the validation cohort based on these features (**Supplementary Figure 3C**). Overall survival analysis indicated a significant survival outcome difference between cluster 1 and cluster 2 in the GBM meta cohort (**Figure 2A**, P = 0.00066), TCGA GBM-array cohort (**Figure 2B**, P = 0.013), and Xiangya cohort (**Figure 2C**, P = 0.0023).

The expression of the LAG difference was also mapped to illustrate its relationship with the cluster model, IDH status, 1p19q status, MGMT methylation status, and glioma CpG island methylator phenotype (G-CIMP) status (**Figures 2D, E**). Most of LAGs, including HIF1A, LDHA, SLC16A1, and SLC16A3, were upregulated in cluster 1 samples, whereas LDHB, PFKFB2, SLC16A7, and SLC16A8 were downregulated (**Supplementary Figure 4A**). Similar expression maps of LDHA, SLC16A1, SLC16A3, LDHB, PFKFB2, SLC16A7, and LSC16A8 were also proved in TCGA GBM array cohort (**Supplementary Figure 4B**). A higher expression was found for HIF1A, LDHA, SLC16A1, SLC16A3, SLC16A8, and UEVLD in IDH wild-type GBM compared with IDH mutant GBM (**Supplementary Figure 4C**). Meanwhile, HIF1A, LDHA, PFKFB2, SLC16A1, SLC16A3, SLC16A8, and UEVLD in 1p19q non-co-deletion GBM had a higher expression relative to 1p19q co-deletion GBM (**Supplementary Figure 4D**). However, differentially expressed genes between MGMT-methylated GBM and unmethylated GBM were LDHA and SLC16A3 (**Supplementary Figure 4E**). As for GCIMP status, a higher expression of LDHA, SLC16A1, SLC16A3, and UEVLD was found to be connected with non G-CIMP GBM and a higher expression of LDHB and SLC16A7 was related to G-CIMP GBM (**Supplementary Figure 4F**). Therefore, dysregulated LAG expression strongly associated with GBM unfavorable clinical features. Considering that LDHA and HIFA promote the production of lactate, and LDHB is responsible for lactate-pyruvate transformation (48), these dysregulated LAGs implied that cluster 1 samples may have severe lactate accumulation than cluster 2 samples.

Furthermore, single-cell RNA-seq analysis was performed to validate the conclusion that lactate may modulate GBM samples’ immunocyte infiltrations (**Figure 2F**). The cluster model was reproduced on single-cell RNA-seq data with the support vector machine algorithm (**Figure 2G**). A previous study identified four cell types of GBM (OPC: oligodendrocyte-progenitor-like, AC: astrocyte-like, NPC: neural-progenitor-like, and MES: mesenchymal-like) based on single-cell RNA-seq analysis (49); GBM with more MES cells possesses poor prognosis. In this work, more MES cells were enriched in cluster 1 samples (**Figure 2H**), proving that cluster 1 consisted of more aggressive growth samples. Taken together, a dysregulated expression of LAGs can be applied to predict GBM prognosis.

### Complicated epigenetic alternations were observed in cluster 1 samples

**Figure 3:**
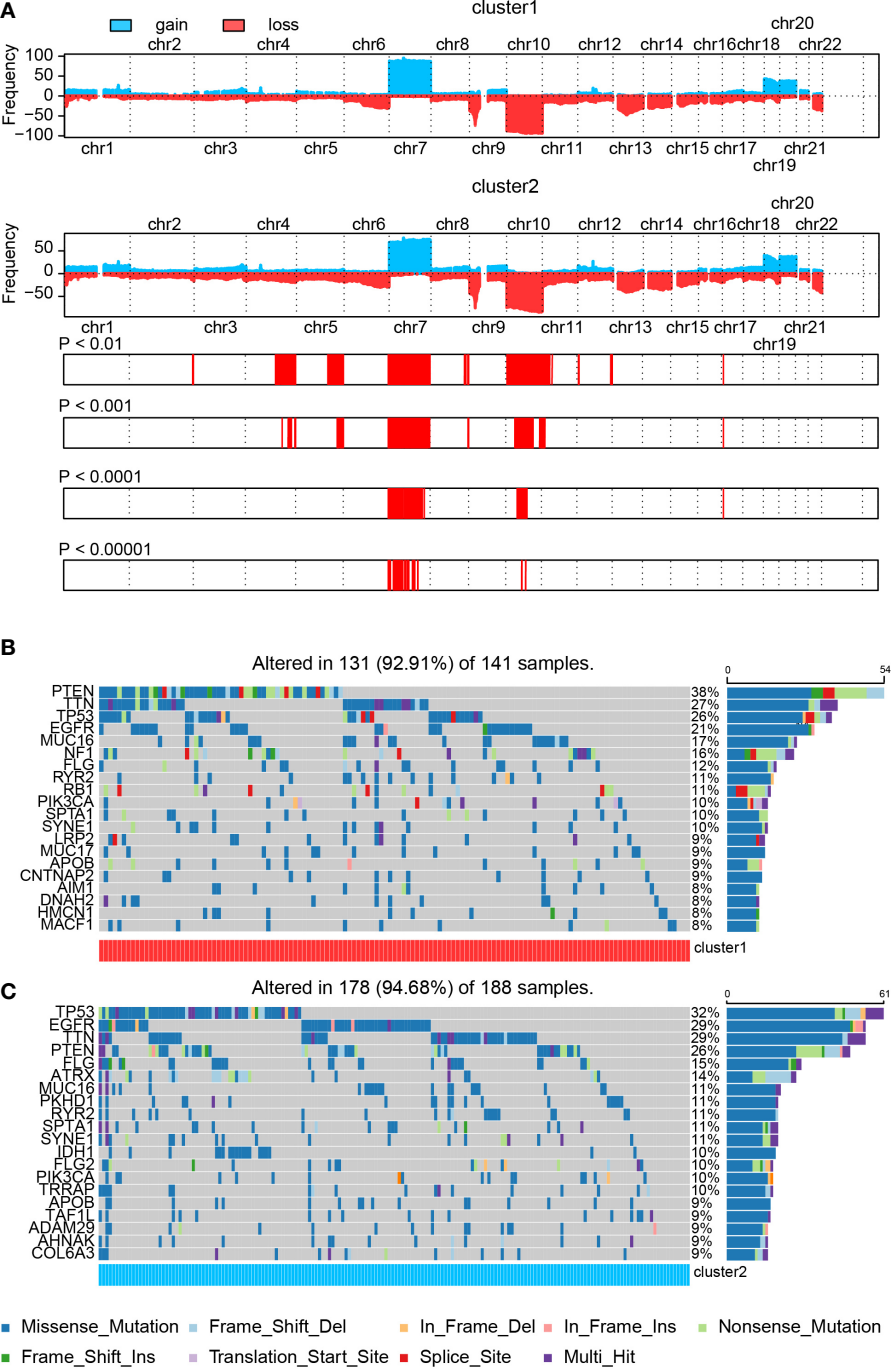
The SNP and CNV difference in the cluster model. **(A)** CNV difference in the cluster model. **(B, C)** SNP difference in the cluster model.

First, comparing the CNV map between cluster 1 and cluster 2 samples, variations were found on chromosomes 3, 4, 5, 7, 8, 9, 10, 11, 12, 13, and 17 (**Figure 3A**). Then, we explored the somatic mutation variation difference between cluster 1 and cluster 2 samples. A somatic mutation signature comparison indicated that only more C > G was noticed in cluster 2 samples than in cluster 1 samples. As for somatic mutation variant classification and type, a significant difference was only observed on frameshift deletion and deletion, respectively (**Supplementary Figure 5A**).

Next, we analyzed the SNP difference between cluster 1 and cluster 2 samples (**Figures 3B, C**). Mutation of PTEN (38% vs. 26%), TTN (27% vs. 29%), TP53 (26% vs. 32%), EGFR (21% vs. 29%), MUC16 (17% vs. 11%), FLG (12% vs. 15%), RYR2 (11% vs. 11%), PIK3CA (10% vs. 10%), SPTA1 (10% vs. 11%), and SYNE1 (10% vs. 11%) was noticed in both cluster 1 and cluster 2 samples. Among these genes, PTEN and MUC16 showed a higher mutation ratio in cluster 1 samples than cluster 2 samples, whereas a high percentage of mutated TP53, EGFR, and FLG was found in cluster 2 samples. In the meantime, mutated NF1 (16% vs. 7%) and RB1 (11% vs. 8%) were significantly enriched in cluster 1 samples. On the other hand, IDH1 (less than 6% vs. 10%), TRRAP (less than 6% vs. 10%), ATRX (less than 6% vs. 14%), PKHD1 (6% vs. 11%), and FLG2 (6% vs. 10%) were noticed in cluster 2 samples. More details about these mutated genes, like IDH1, ATRX, and NF1, are summarized in **Supplementary Figure 5B**.

A correlation analysis of top 25 mutated genes in cluster 1 and cluster 2 samples was conducted (**Supplementary Figures 5C, D**). Co-occurrence pairs like FCGBP–LRP2 and FAT4–SYNE1 were noticed in cluster 1 samples. Meanwhile, pairs like RELN–DNAH9, LRP2–AHNAK, DNAH9–SYNE1, AHNAK–SYNE1, and ADAM29–SYNE1 were found in cluster 2 samples. Interestingly, two exclusive pairs, PIK3CA–PTEN and IDH1–PTEN, were also discovered in cluster 2 samples. Mutated PTEN, PIK3CA, ATRX, and IDH1 have been proved to be associated with GBM prognosis (50–52). Other mutated genes like FAT4 (53), SYNE1 (54), and ADAM29 (55) have been proved to be associated with tumor prognosis but not with GBM. Taken together, those novel mutated genes may assist in predicting GBM patients’ prognosis.

### Higher tumor lactate levels affect the tumor immune microenvironment

**Figure 4:**
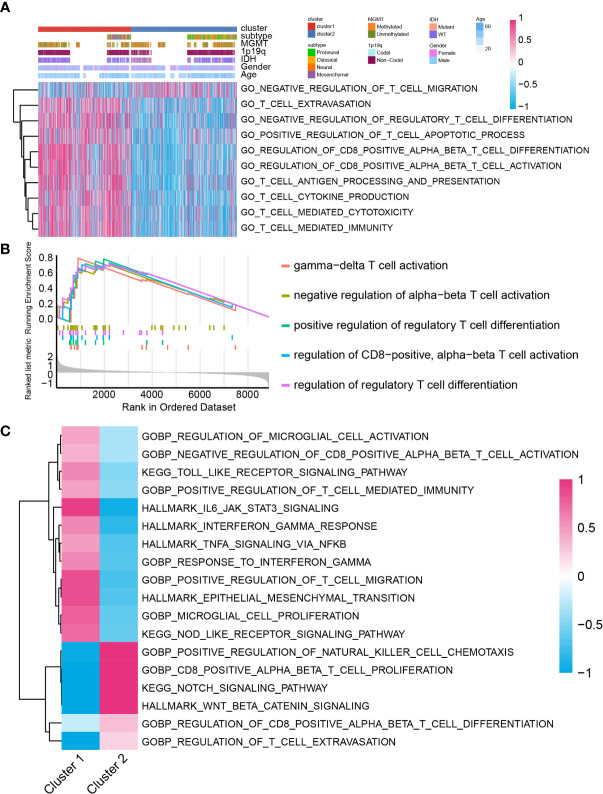
The biofunction prediction based on the cluster model in the GBM meta cohort. Biofunction prediction based on GSVA **(A)** and GSEA **(B)**. **(C)** GSEA enrichment based on single cell RNA-seq data.

Several studies proposed that lactate can modulate immunocyte function, activation, and infiltration (10–16). Therefore, we investigated the immunocyte infiltration ratio difference between cluster 1 and cluster 2. Biofunction prediction based on GSVA and GSEA indicated that T-cell migration, activation, extravasation, and differentiation-associated pathways were selectively activated in cluster 1 and cluster 2 samples (**Figures 4A, B**). Moreover, pathway T-cell cytokine production, T cell-mediated cytotoxicity, T cell-mediated immunity, and T-cell antigen processing and presentation were also preferentially activated in cluster 1 samples than in cluster 2 samples. In the meantime, validation on TCGA GBM array database indicated similar conclusions (**Supplementary Figures 6A, B**). In single-cell RNA-seq analysis, biofunction prediction based on GSEA also suggested that T cell-related pathways were enriched in cluster 1 samples (**Figure 4C**).

### High-lactate secretion may contribute to an immunosuppressive microenvironment

**Figure 5:**
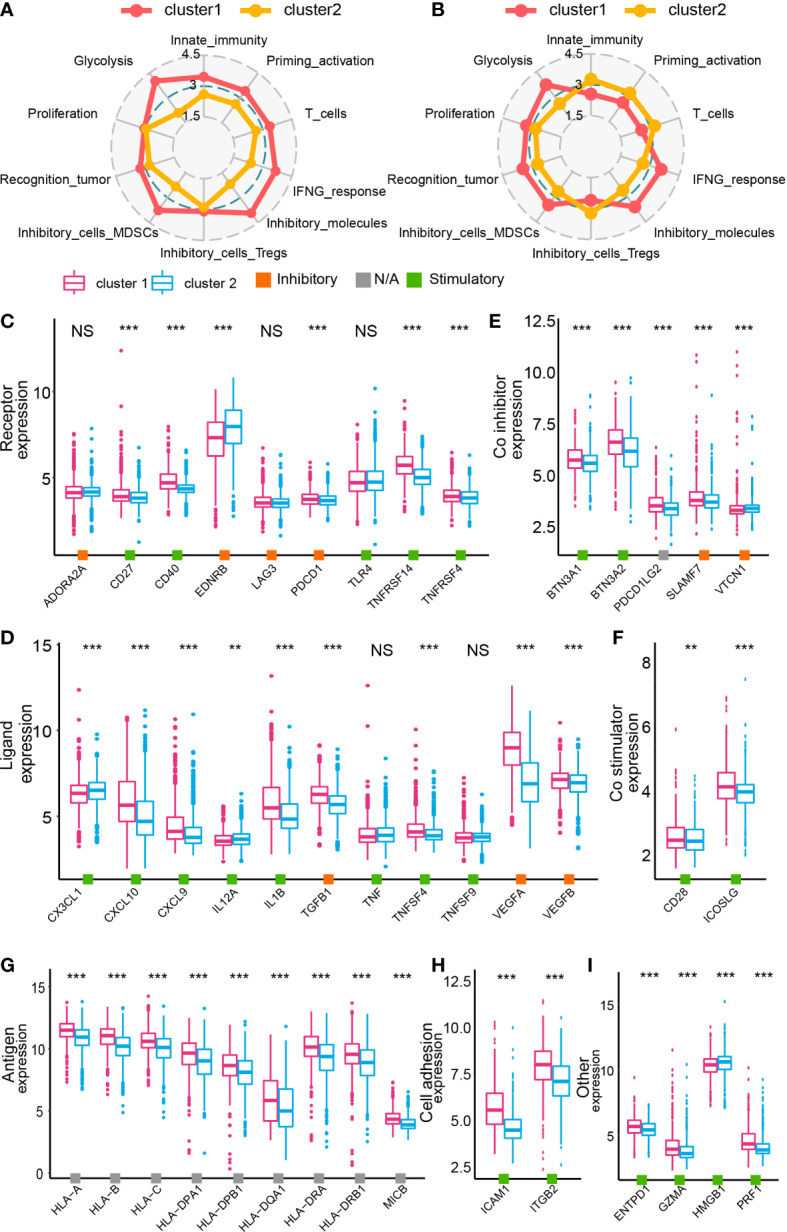
The immune landscape of cluster 1 and cluster 2 samples from the GBM meta cohort. Immunogram based on the GBM meta cohort **(A)** and data from single-cell RNA-seq analysis **(B)**. The expression of immune inhibitors/contributors, including receptor **(C)**, ligand **(D)**, co-inhibitor **(E)** co-stimulator **(F)**, antigen **(G)**, cell adhesion **(H)**, and other **(I)**. NS, no significance, **P < 0.01, ***P < 0.001.

A previous study proposed 10 characteristics of tumor immune landscape (32). In bulk RNA-seq analysis, cluster 1 samples scored higher in glycolysis, innate immunity, priming activation, T cells, IFNG response, inhibitory molecules, marrow-derived suppressor cells, and recognition of tumor cells (GBM meta cohort in **Figure 5A** and **Supplementary Figure 9A**, TCGA GBM array cohort in **Supplementary Figures 7A**, **9B**, Xiangya cohort in **Supplementary Figures 8A**, **9C**). Nevertheless, after focusing on tumor cells from single-cell RNA-seq analysis, tumor cells from cluster 1 had higher scores on glycolysis, proliferation, recognition of tumor cells, marrow-derived suppressor cells, inhibitory molecules, and IFNG response, whereas cluster 2 tumor cells had higher scores on innate immunity, priming and activation, T cells, and regulatory T cells (**Figure 5B** and **Supplementary Figure 9D**). Although cluster 1 samples had higher scores on recognition of tumor cells, immunosuppressor features like marrow-derived suppressor cells, inhibitory molecules, and IFNG response were enriched in cluster 1 samples. Correspondingly, immunity activation-associated features were active in cluster 2 samples. Together, GBM cells in cluster 1 samples may inhibit immunity activation and contribute to an immunosuppressive microenvironment.

The expression map of immunomodulators, including receptor, ligand, co-inhibitor, co-stimulator, antigen, cell adhesion molecules, and other genes, was depicted based on the cluster model (38). According to bulk RNA-seq analysis, most of modulators were upregulated in cluster 1 samples (GBM meta cohort in **Figures 5C–I**, TCGA GBM array cohort in **Supplementary Figures 7B, H**, Xiangya cohort in **Supplementary Figures 8B, H**). However, results from the single-cell RNA-seq analysis suggested that even if a dysregulated expression of those modulators was also found, not all of them were upregulated in cluster 1 samples (**Supplementary Figure 10**). Therefore, to further specify the role of those modulators in cell–cell communication and the immunosuppressive microenvironment, we predicted the interactions between tumor cells and other cell types.

### High lactate level affects CD8 T-cell infiltration

**Figure 6:**
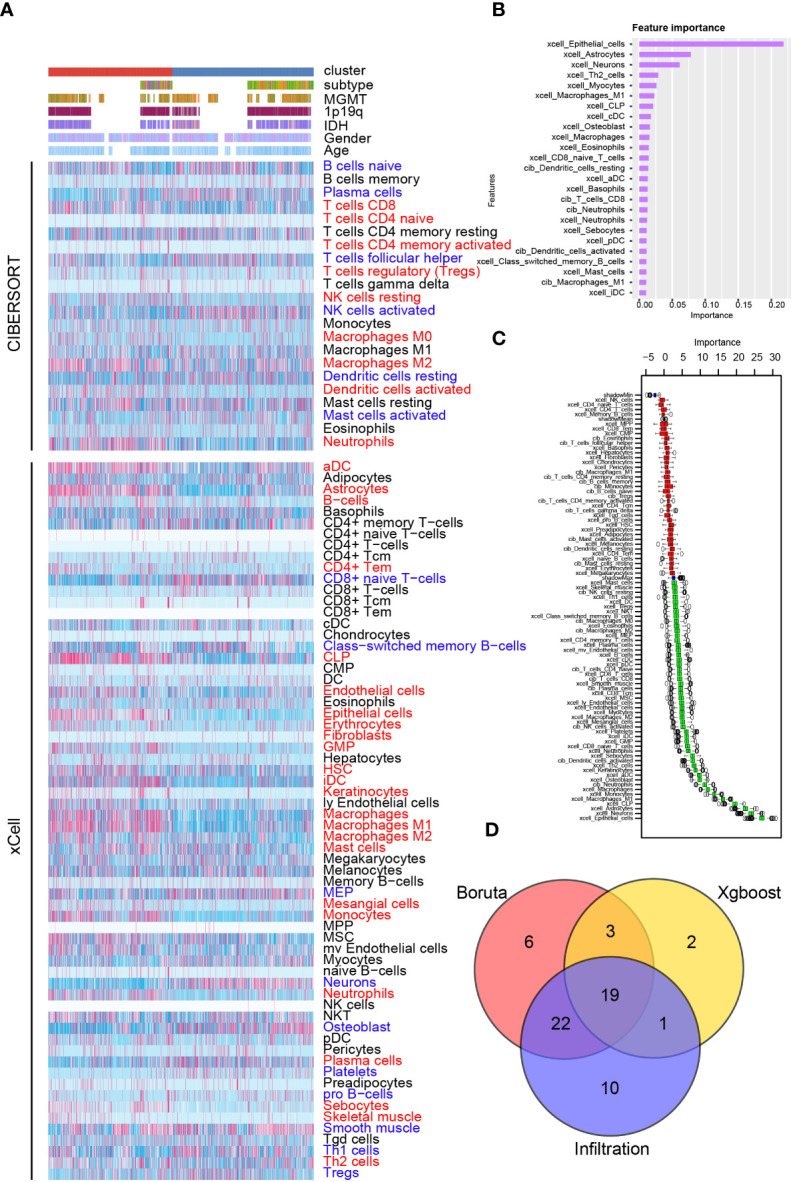
Immunocyte infiltration difference between cluster 1 and cluster 2. **(A)** Immunocyte infiltration analysis based on CIBERSORT and xCell algorithm. Immunocyte characteristic difference based on the cluster model using machine algorithm “Xgboost” **(B)** and “Boruta” **(C)**. **(D)** Interaction of immunocyte infiltration and results from “Xgboost” and “Boruta”.

Therefore, we investigated the tumor immune landscape difference between cluster 1 and cluster 2 samples. Results from the ESTIMATE algorithm suggested that cluster 1 samples had a higher immune score, higher stromal score, and lower tumor purity, implying a more complicated immune landscape in cluster 1 than cluster 2 samples (**Supplementary Figures 11A, B**). CIBERSORT and xCell algorithm were introduced to analyze the immunocyte infiltration ratio difference between cluster 1 and cluster 2 samples from the training cohort (**Figure 6A** and **Supplementary Figure 12**) and the validation cohort (**Supplementary Figures 11C**, **12**). In cluster 1, CD8 T cells, CD4 T cells, activated dendritic cells, and macrophages were preferentially infiltrated. Meanwhile, a higher infiltration ratio of B cells, resting dendritic cells, and Th1 cells was observed in cluster 2 samples. Then, two machine learning algorithms, XGboost and Boruta, were used to identify the cluster model-associated immunocytes (**Figures 6B, C**). A higher importance score represents a stronger relationship with cluster 1 samples. As illustrated, stromal cells like epithelial cells, astrocytes, and neurons and immunocytes like Th2 cells, M1 macrophages, CD8 T cells and dendritic cells were recognized as cluster 1 samples’ characteristic. Integrating results from immunocyte infiltration ratio analysis, the Xgboost and the Boruta algorithm, CD8 T cells, dendritic cells, and macrophages were selected and considered as cluster 1 sample-associated immunocytes (**Figure 6D**).

### Lactate modulates CD8 T cells, microglia, and macrophage

A cell–cell interaction within various cell types was analyzed, and their role in sending or receiving signals was illustrated (**Supplementary Figures 13A, B**). Then, several potential differentially activated pathways between tumor cells and other cells were selected, including IL17 signaling pathway (**Supplementary Figure 13C**), IFN-II signaling pathway (**Supplementary Figure 13D**), VEGF signaling pathway (**Supplementary Figure 13E**), PERIOSTIN signaling pathway (**Supplementary Figure 13F**), TWEAK signaling pathway (**Supplementary Figure 13G**), and PAR signaling pathway (**Supplementary Figure 13H**).

**Figure 7:**
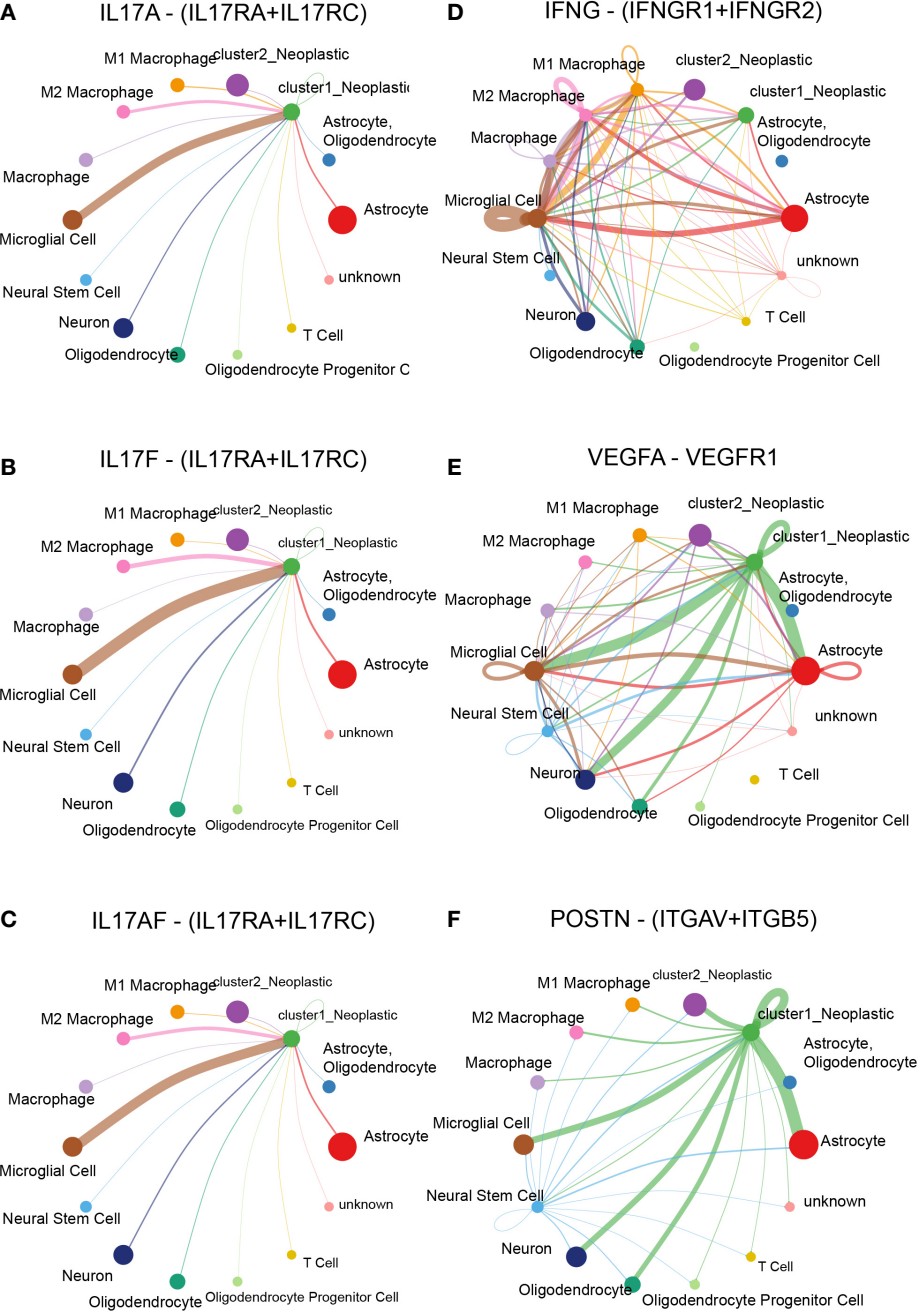
Cell–cell interaction based on the cluster model in single-cell RNA-seq analysis. Ligand–receptor pair difference between cluster 1 and cluster 2 samples, including IL17A-(IL17RA+IL17RC) **(A)**, IL17F-(IL17RA+IL17RC) **(B)**, IL17AF-(IL17RA+IL17RC) **(C)**, IFNG-(IFNGR1+IFNGR2) **(D)**, VEGFA-VEGFR1 **(E)**, and POSTN-(ITGAV+ITGB5) **(F)**.

**Figure 8:**
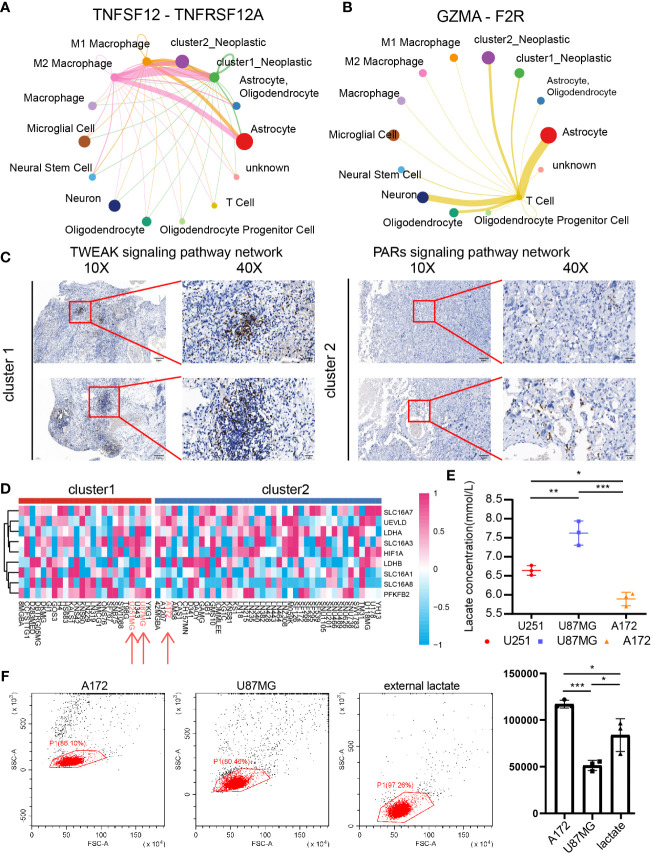
The role of CD8+ T cells in cluster 1 and cluster 2 samples. Cluster 1 and cluster 2 tumor cell communication with macrophage and CD8+ T cells through TNFSF12-TNFRSF12A **(A)** and GZMA-F2R **(B)**, respectively. **(C)** IHC revealed the CD8+ T-cell infiltration difference between cluster 1 and cluster 2 samples. **(D)** The cluster model in central nervous system tumor cell lines. **(E)** Lactate concentrations in U251MG, U87MG, and A172. N = 3. *P < 0.05, **P < 0.01, ***P < 0.001. **(F)** Percentage of migrated of CD8+ T cells after coculture with U87MG and A172 for 48 h. N = 3. *P < 0.05, **P < 0.01, ***P < 0.001.

Specifically, ligand–receptor pairs, such as IL17A-(IL17RA+IL17RC) (**Figure 7A**), IL17F-(IL17RA+IL17RC) (**Figure 7B**), IL17AF-(IL17RA+IL17RC) (**Figure 7C**), IFNG-(IFNGR1+IFNGR2) (**Figure 7D**), VEGFA-VEGFR1 (**Figure 7E**), POSTN-(ITGAV+ITGB5) (**Figure 7F**), TNFSF12-TNFRSF12A (**Figure 8A**), and GZMA-F2R (**Figure 8B**), were identified, suggesting that tumor cells with different lactate secretions may modulate immunocytes through these pairs.

Compared with cluster 2 tumor cells, cluster 1 tumor cells communicated with microglia more actively through the IL17 signaling pathway (**Figures 7A–C**), the IFN-II signaling pathway (**Figure 7D**), the VEGF signaling pathway (**Figure 7E**), and the PERIOSTIN signaling pathway (**Figure 7F**). In addition, cluster 1 tumor cells also received more signals from periphery blood M1 and M2 macrophage than cluster 2 tumor cells (**Figure 8A**). Interestingly, cluster 2 tumor cells received more signals from T cells than cluster 1 tumor cells (**Figure 8B**). Together, those results implied that lactate may affect T cells, microglia, and periphery blood macrophage.

Integrating results from biofunction prediction, we explored the potential effect of lactate on CD8 T cells. The infiltration ratio of CD8+ T cells in cluster 1 and cluster 2 samples was first validated. We selected one sample from each cluster based on the Xiangya cohort and further examined the infiltration ratio of CD8 T cells and IHC. As indicated, the ratio in in cluster 1 was higher than in cluster 2 (**Figure 8C**). Then, we focused on the influence of tumor with different lactate production abilities on CD8+ T cells. Central nervous system tumor cell lines’ expression was obtained from CCLE, and the support vector machine algorithm was performed on group cell lines. GBM cell lines U87MG and U251MG were grouped into cluster 1, whereas GBM cell line A172 was labeled as cluster 2 (**Figure 8D**). As predicted, U251MG (6.642 ± 0.128 mmol/l) and U87MG (7.624 ± 0.317 mmol/l) had high lactate concentrations in cultural medium than A172 (5.891 ± 0.178 mmol/l) (**Figure 8E**). Then, we exposed CD8 T cells to GBM cells (U87MG and A172) or external lactate (7.5 mmol/l) to inquire if lactate concentration can affect CD8 T-cell migration. As illustrated, the low lactate concentration group (A172 group: 117,109.333 ± 4,217.798; external lactate group: 83,844.667 ± 17,576.598) recruits more CD8 T cells than the high lactate concentration group (U87MG group: 51,327.333 ± 5,442.792) (**Figure 8F**). Along with recent discovery (56), lactate can increase stemness of CD8 T-cell in colon cancer model. However, lactate can also weak CD4 T cell and CD8 T cell motility in inflammatory sites (57) indicating the complicate role of lactate in regulating CD8 T cells in GBM.

### Novel chemo-compound treatment strategy for glioblastoma with different lactate secretion abilities

**Figure 9:**
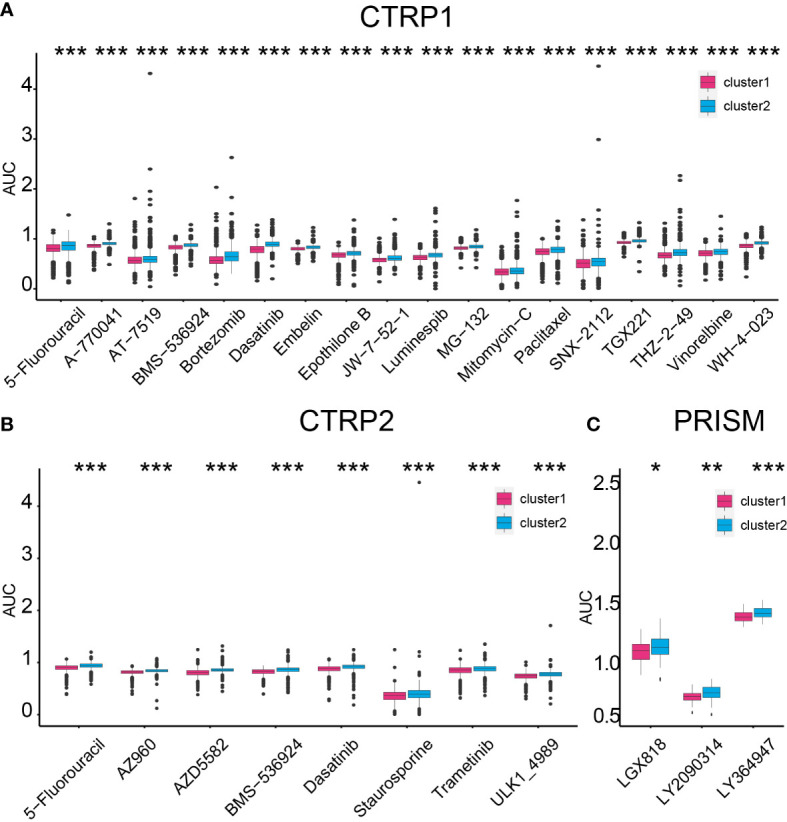
Compound prediction based on the cluster model. Cluster 1 sample sensitive compounds from CTRP1 **(A)**, CTRP2 **(B)**, and PRISM **(C)** databases. *P < 0.05, **P < 0.01, ***P < 0.001.

Based on two clusters, potential sensitive drugs from the CTRP1, CTRP2, and PRISM databases were predicted. Cluster 1 samples showed higher sensitivity to 5-fluorouracil, A-770041, AT-7519, BMS-536924, bortezomib, dasatinib, embelin, epothilone B, JW-7-52-1, luminespib, MG-132, mitomycin-C, paclitaxel, SNX-2112, TGX221, THZ-2-49, vinorelbine, WH-4-023, AZ960, AZD5582, staurosporine, trametinib, ULK1-4989, LGX818, LY2090314, and LY364947 (**Figures 9A–C**).

On the contrary, cluster 2 samples were sensitive to ACY-1215, AR-42, CI-1033, CUDC-101, CX-5461, GSK1059615, GSK690693, OSI-027, PF-00299804, PI-103, QL-X-138, WYE-125132, ABT737, acetalax, dinaciclib, sabutoclax, vincristine, I-BET151, and indisulam (**Supplementary Figures 14A, C**). Thus, selecting proper compounds may assist in targeting tumors with different lactate secretion abilities.

## Discussion

GBM can be classified into three subtypes (5, 6), namely, proneural, classical, and mesenchymal, and following studies identified that mesenchymal GBM had worse prognosis relative to other subtypes. Proneural–mesenchymal transition was related to GBM sensitivity to treatments, like radiotherapy and chemotherapy, recurrence, and progression. Therefore, recent years’ several studies proposed that targeting proneural–mesenchymal transition may slow GBM progression. In this work, we constructed a cluster model based on LAGs, and GBM samples were classified into different groups, cluster 1 and cluster 2. Following exploration identified that cluster 1 manifested worse clinical outcomes and higher lactate secretion ability than cluster 2 samples. Moreover, cluster 1 samples also consisted of a high proportion of MES-like cells, which is also a characteristic of MES GBM, implying its inner relationship with GBM subtypes (49), according to single-cell RNA-seq analysis. Therefore, interfering with tumor lactate secretion ability may improve GBM prognosis by modulating proneural–mesenchymal transition.

Abnormal LAG expressions have been widely explored in GBM (58, 59). In this work, pan-cancer analysis suggested that methylation, SNP, and CNV did not affect LAG expression in GBM as much as in other tumor types like lung cancer or breast cancer. Interestingly, a recent theory proposed that the generation of mesenchymal GBM may result from dysregulated transcriptional activity (1). Studies observed that GBM with a high glycolysis rate, which means higher lactate secretion ability, was associated with extensive transcription factor network alternation (60). Since HIF1α is a critical transcription factor and several studies reported its constant expression under non-hypoxic circumstance (61–63), the high lactate secretion ability of GBM may also result from abnormal regulation of the transcription factor network. Nevertheless, this hypothesis still requires massive experiments to support and validate.

Several potential chemical compounds that target tumors with different lactate secretion abilities were predicted. For instance, 5-fluorouracil (64, 65), bortezomib (66), trametinib (67), and LY2090314 (68) can affect glycolysis to inhibit tumor progression. As cluster 1 samples are more sensitive to these compounds and have higher lactate secretion ability than cluster 2 samples, targets to tumor based on their characteristics can assist in inhibiting tumor progression.

An immunosuppressive microenvironment in GBM leads to tumor poor response to immunotherapy (1). Since higher infiltration of microglia (23), macrophage (25), and CD8 T cells (20, 69) can contribute to a tumor immunosuppressive microenvironment, we found that lactate accumulation may be one potential way to determine how GBM affects these immunocytes. For instance, cluster 1 samples interact with microglia through IL17, VEGF, and POSTN, or with macrophage through TNFSF12, or with CD8 T cells through F2R more active than cluster 2 samples. IL17 can activate microglia (70, 71) and promote GBM progression (72, 73). The connection between POSTN and regulating TGF-β1, HIF-1α, and VEGFA expression has been confirmed, which can affect GBM progression and macrophages (74, 75). TNFSF12 has been confirmed to modulate tumor progression through interacting with macrophage (76, 77). Therefore, future exploration on the relationship between tumor cells and immunocytes through these ligand–receptor pairs may offer insights on understanding the failure of GBM immunotherapy and GBM immunosuppressive microenvironment.

In an *in vitro* validation, cluster 1 cell lines produced more lactate relative to cluster 2 cell lines. Meanwhile, more CD8+ T cells infiltrated in cluster 1 samples in tumor samples, but the ability of CD8+ T cells’ migration was inhibited when cultured with highlactate produced tumor cells. A recent work proposed that high lactate can increase the population of stem-like CD8+ T cells in colon cancer model, but another study found that CD8+ T cell motility is inhibited in high lactate environment (57) which may explain the inhibition on CD8 T cell migration ability in vitro in our finding. Regarding to the effect of lactate on CD8 T cell, in acute myeloid leukemia, elevated lactate levels dysregulated CD8+ T-cell function (78). Decreasing extracellular lactate levels can not only transform M2 macrophage into M1 macrophage but also restore CD8+ T-cell activity (79). Meanwhile, lactate was also reported to obliterate CD8+ T-cell function, which results in tumor poor response to immunity (80–83). Therefore, although high-infiltration CD8+ T cells were noticed in cluster 1 samples, high lactate accumulation may also suppress the normal function of CD8+ T cells and result in a tumor immunosuppressive microenvironment.

Tumor response to the IFNG difference between cluster 1 and cluster 2 samples was identified, and it was also predicted as one possible reason that contributes to GBM malignancy. The role of IFNG in GBM became more and more significant in recent years’ research, including GBM progression and the tumor immunosuppressive microenvironment. Previous IFNG-related gene signature models predicted the prognosis of GBM and its sensitivity to immunotherapy and radiotherapy (84), implying its significant role in GBM. Moreover, high production of IFNG by T cells can promote upregulation of PD-L1 in tumor cells, which will weaken the tumor response to CAR-T cells (85). In a mouse model bearing intracranial tumor treated with temozolomide, IFNG-produced CD8+ T cells prolonged mouse survival time than IL17-produced CD8+ T cells and IFNG/IL17-produced CD8+ T cells (73). Inhibition on the IFNG-triggered JAK/STAT pathway can prevent glioma invasion and tumorigenesis (86). Therefore, high IFNG can promote GBM progression and weaken tumor response to immunotherapy. In this work, cluster 1 samples showed a higher response to IFNG, also indicating the significant role of IFNG in GBM progression.

In summary, the cluster model based on lactate expression can predict GBM progression, and MES-like cells may secrete more lactate. Moreover, high lactate accumulation in a GBM microenvironment also contributes to an immunosuppressive microenvironment. Higher immune inhibitor-associated genes like LAGs, VEGF, EDNRB, and TGFB1 were also upregulated, implying the critical role of lactate in a tumor immunosuppressive microenvironment. Candidate compounds targeted to cluster 1 sample prediction were predicted. For instance, bortezomib in combination with temozolomide can not only prolong GBM patients’ survival time but also improve tumor immunological response (87). Vinorelbine can activate stem-like CD8+ T cells and improve anti-PD-1 therapy efficiency in breast cancer (88). Treating lung cancer with trametinib can increase NK-cell and T-cell infiltration and elevate tumor response to immunotherapy (89). Hence, those compounds may assist in GBM immunotherapy.

## Data availability statement

Data used in this work can be acquired from the GEO, the TCGA, and the CGGA database and so on. Other datasets generated in the current study could be available by contacting the corresponding author.

## Ethics statement

This study was reviewed and approved by the ethics committee of Xiangya Hospital, Central South University.

## Author contributions

Writing—original draft: ZYW. Writing—review and editing: ZYW and HZ. Data curation: ZYW, ZD, and HZ. Formal analysis: ZYW and ZD. Validation: XL and XZ. Visualization: ZYW, JZ, and ZL. Methodology: ZYW, ZPW, and PL. Funding acquisition: MYZ and QC. Project administration: QC. Supervision: MYZ and QC. All authors contributed to the article and approved the submitted version.
