# Supplementary material for: Tumor-secreted lactate contributes to an immunosuppressive microenvironment and affects CD8 T-cell infiltration in glioblastoma
Source: Front Immunol. 2023 Apr 12;14:894853. doi: 10.3389/fimmu.2023.894853 (PMC10130393; doi:10.3389/fimmu.2023.894853)
Supplement: Supplementary file 1 [file Presentation_1.pdf]

Supplementary Figure 1| Correlation between methylation, CNV and LAGs expression. (A) The spearman correlation between LAGs methylation and its expression. (B) The correlation between LAGs CNVs and its expression.

Supplementary Figure 2| Homozygous/heterozygous amplification and deletion of LAGs in different tumor types. (A) The homozygous amplification and deletion of LAGs. (B) The heterozygous amplification and deletion of LAGs.

Supplementary Figure 3| Details about the cluster model. (A) Principal Component Analysis based on LAGs. (B) Details about the cluster model. (C) Characteristics identified by the support vector machine.

Supplementary Figure 4| The cluster model based on LAGs and LAGs expression. (A) LAGs expression difference in the cluster model in GBM meta cohort. (B) LAGs expression difference in the cluster model in TCGA GBM array cohort. (C) LAGs expression difference in the IDH status in GBM meta cohort. (D) LAGs expression difference in the 1p19q status in TCGA GBM array cohort. (E) LAGs expression difference in the MGMT status in GBM meta cohort. (F) LAGs expression difference in the G-CIMP status in TCGA GBM array cohort.

Supplementary Figure 5| SNV and SNP different between cluster 1 and cluster 2. (A) Somatic mutation signatures comparison between cluster 1 and cluster 2 samples. (B) Details about SNPs in the cluster model. (C-D) Correlation analysis of top 25 mutated genes in cluster 1 and cluster 2 samples was conducted respectively.

Supplementary Figure 6| The biofunction prediction based on the cluster model in TCGA GBM array cohort. Biofunction prediction based on GSVA (A) and GSEA analysis (B).

Supplementary Figure 7| The immune landscape of cluster 1 and cluster 2 samples from TCGA GBM array cohort. (A) Immunogram based on TCGA GBM array cohort. The expression of immune inhibitors/contributors, including antigen (B), receptor (C), ligand (D), other (E), cell adhesion (F), co-inhibitor (G) and co-stimulator (H).

Supplementary Figure 8| The immune landscape of cluster 1 and cluster 2 samples from Xiangya cohort. (A) Immunogram based on Xiangya cohort. The expression of immune inhibitors/contributors, including antigen (B), receptor (C), ligand (D), other (E), cell adhesion (F), co-inhibitor (G) and co-stimulator (H).

Supplementary Figure 9| Details about the immunogram. Score of each component of immunogram based on GBM meta cohort (A), TCGA GBM array cohort (B), Xiangya cohort (C) and single cell RNA-seq analysis (D).

Supplementary Figure 10| The expression of immune inhibitors/contributors in the cluster model based on single cell RNA-seq analysis. The expression of immune inhibitors/contributors, including receptor (A), ligand (B), antigen (C), co-inhibitor (D) co-

stimulator (E), cell adhesion (F) and other (G).

Supplementary Figure 11| Details about immunocytes infiltration analysis. Immunocytes infiltration analysis based on ESTIMATE algorithm in GBM meta cohort (A) and TCGA GBM array cohort (B). (C) Immunocytes infiltration analysis based on CIBERSORT and xCell algorithm in TCGA GBM array cohort.

Supplementary Figure 12| Details about immunocytes infiltration analysis. Results from CIBERSORT algorithm in the GBM meta cohort (A) and TCGA GBM array cohort (B). Results from xCell algorithm in the GBM meta cohort (C) and TCGA GBM array cohort (D).

Supplementary Figure 13| Details about cell-cell interaction. (A-B) The role of different cells acts during cell-cell interaction. Different communication between cluster 1 and cluster 2 cells, including IL17 signaling pathway (C), IFN-II signaling pathway (D), VEGF signaling pathway (E), PERIOSTIN signaling pathway (F), TWEAK signaling pathway (G) and PARs signaling pathway (H).

Supplementary Figure 14| Compounds prediction based on the cluster model. Cluster 2 samples sensitive compounds from CTRP1 (A), CTRP2 (B) and PRISM (C) database.

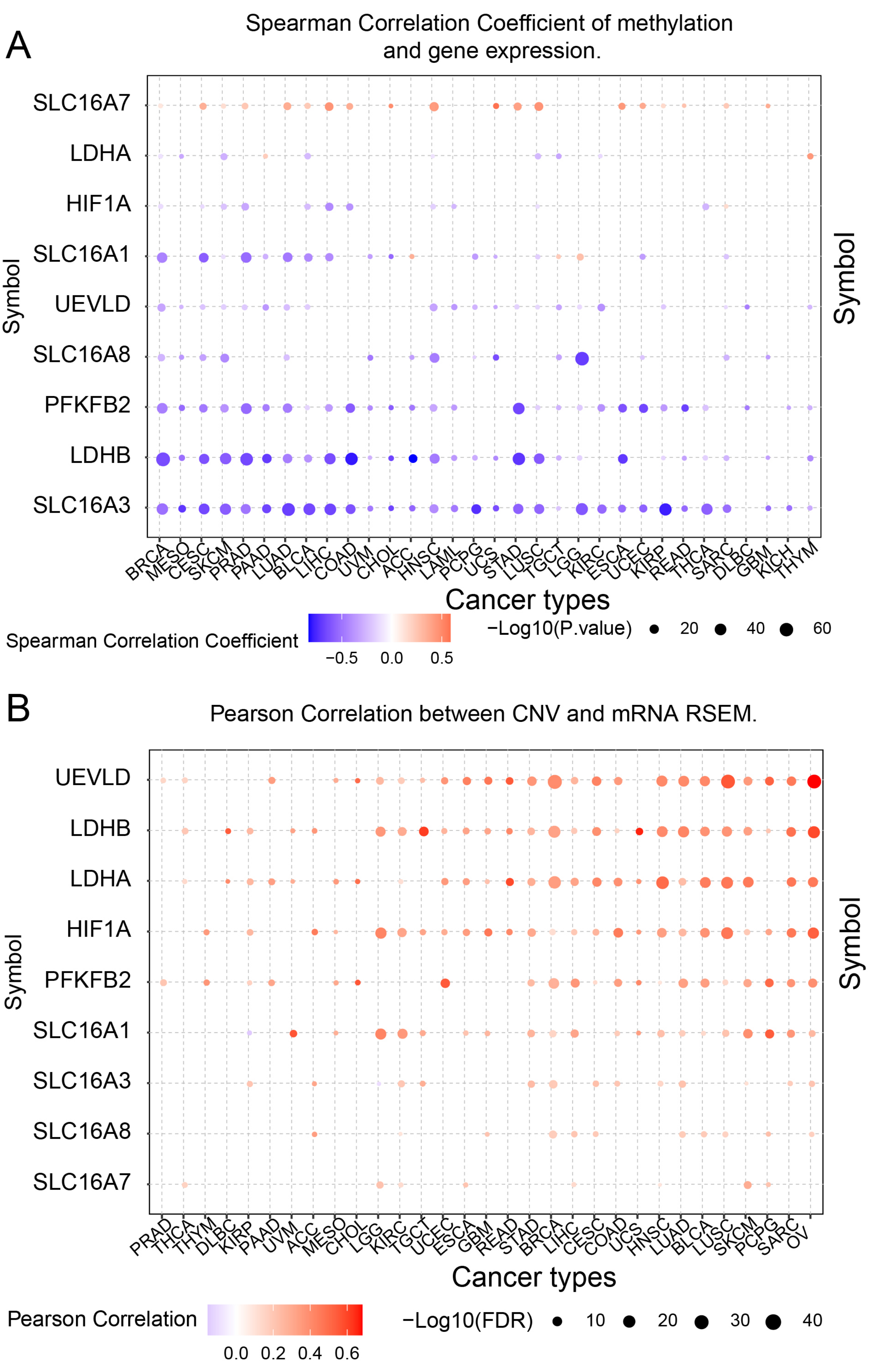
A
Spearman Correlation Coefficient of methylation and gene expression.
SLC16A7
LDHA
HIF1A
SLC16A1
UEVLD
SLC16A8
PFKFB2
LDHB
SLC16A3
Symbol
BRCA MESO CESC SKCM PRAD PAAD LUAD BLCA LIHC COAD UVM CHOL ACC HNSC LAML PCPG UCS STAD LUSC TGCT LGG KIRC ESCA UCEC KIRP READ THCA SARC DLBC GBM KICH THYM
Cancer types
Spearman Correlation Coefficient
−0.5 0.0 0.5
−Log10(P.value) 20 40 60
B
Pearson Correlation between CNV and mRNA RSEM.
UEVLD
LDHB
LDHA
HIF1A
PFKFB2
SLC16A1
SLC16A3
SLC16A8
SLC16A7
Symbol
PRAD THCA THYM DLBC KIRP PAAD UVM ACC MESO CHOL LGG KIRC TGCT UCEC ESCA GBM READ STAD BRCA LIHC CESC COAD UCS HNSC LUAD BLCA LUSC SKCM PCPG SARC OV
Cancer types
Pearson Correlation
0.0 0.2 0.4 0.6
−Log10(FDR) 10 20 30 40

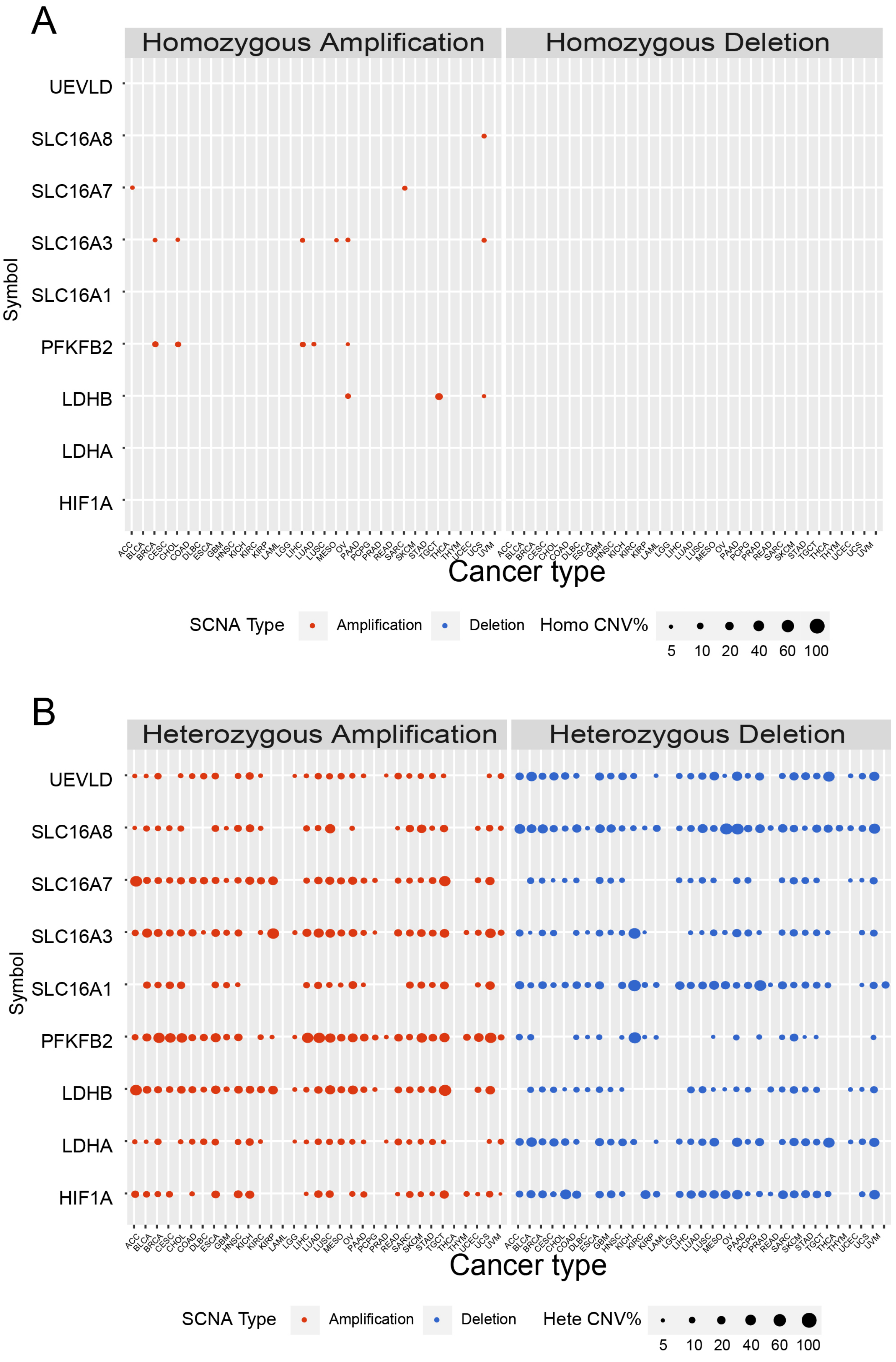
A
Homozygous Amplification
Homozygous Deletion
UEVLD
SLC16A8
SLC16A7
SLC16A3
SLC16A1
PFKFB2
LDHB
LDHA
HIF1A
Symbol
ACC BLCA BRCA CESC CHOL COAD DLBC ESCA GBM HNSC KICH KIRC KIRP LAML LGG LIHC LUAD LUSC MESO OV PAAD PCPG PRAD READ SARC SKCM STAD TGCT THCA THYM UCEC UCS UVM
Cancer type
SCNA Type
Amplification
Deletion
Homo CNV%
5 10 20 40 60 100
B
Heterozygous Amplification
Heterozygous Deletion
UEVLD
SLC16A8
SLC16A7
SLC16A3
SLC16A1
PFKFB2
LDHB
LDHA
HIF1A
Symbol
ACC BLCA BRCA CESC CHOL COAD DLBC ESCA GBM HNSC KICH KIRC KIRP LAML LGG LIHC LUAD LUSC MESO OV PAAD PCPG PRAD READ SARC SKCM STAD TGCT THCA THYM UCEC UCS UVM
Cancer type
SCNA Type
Amplification
Deletion
Hete CNV%
5 10 20 40 60 100

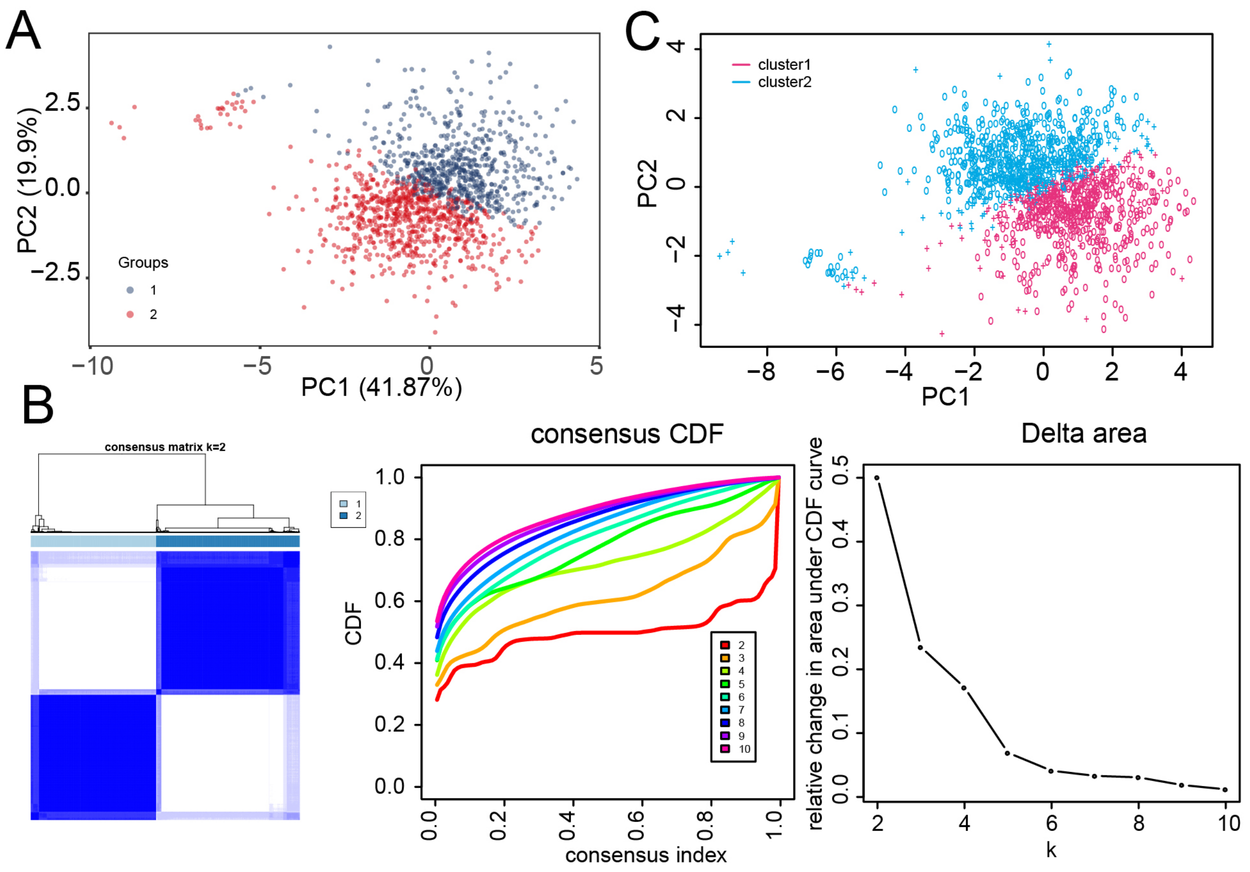

A
PC2 (19.9%)
PC1 (41.87%)
Groups
1
2
B
consensus matrix k=2
1
2
consensus CDF
CDF
consensus index
2
3
4
5
6
7
8
9
10
Delta area
relative change in area under CDF curve
k
C
cluster1
cluster2
PC2
PC1

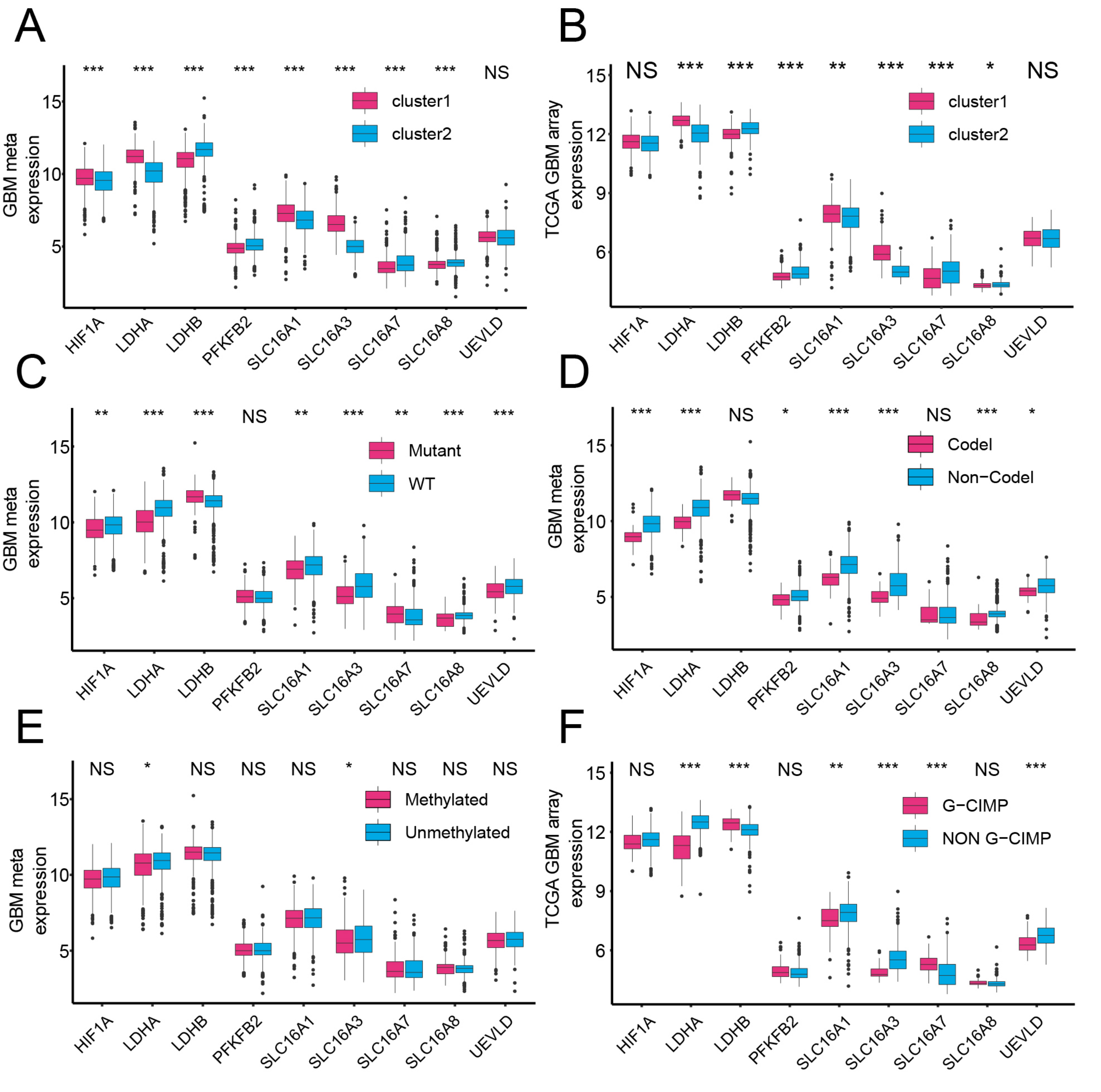

A
GBM meta expression
*** *** *** *** *** *** *** *** NS
cluster1
cluster2
HIF1A LDHA LDHB PFKFB2 SLC16A1 SLC16A3 SLC16A7 SLC16A8 UEVLD
B
TCGA GBM array expression
NS *** *** *** ** *** *** * NS
cluster1
cluster2
HIF1A LDHA LDHB PFKFB2 SLC16A1 SLC16A3 SLC16A7 SLC16A8 UEVLD
C
GBM meta expression
** *** *** NS ** *** ** *** ***
Mutant
WT
HIF1A LDHA LDHB PFKFB2 SLC16A1 SLC16A3 SLC16A7 SLC16A8 UEVLD
D
GBM meta expression
*** *** NS * *** *** NS *** *
Codel
Non−Codel
HIF1A LDHA LDHB PFKFB2 SLC16A1 SLC16A3 SLC16A7 SLC16A8 UEVLD
E
GBM meta expression
NS * NS NS NS * NS NS NS
Methylated
Unmethylated
HIF1A LDHA LDHB PFKFB2 SLC16A1 SLC16A3 SLC16A7 SLC16A8 UEVLD
F
TCGA GBM array expression
NS *** *** NS ** *** *** NS ***
G−CIMP
NON G−CIMP
HIF1A LDHA LDHB PFKFB2 SLC16A1 SLC16A3 SLC16A7 SLC16A8 UEVLD

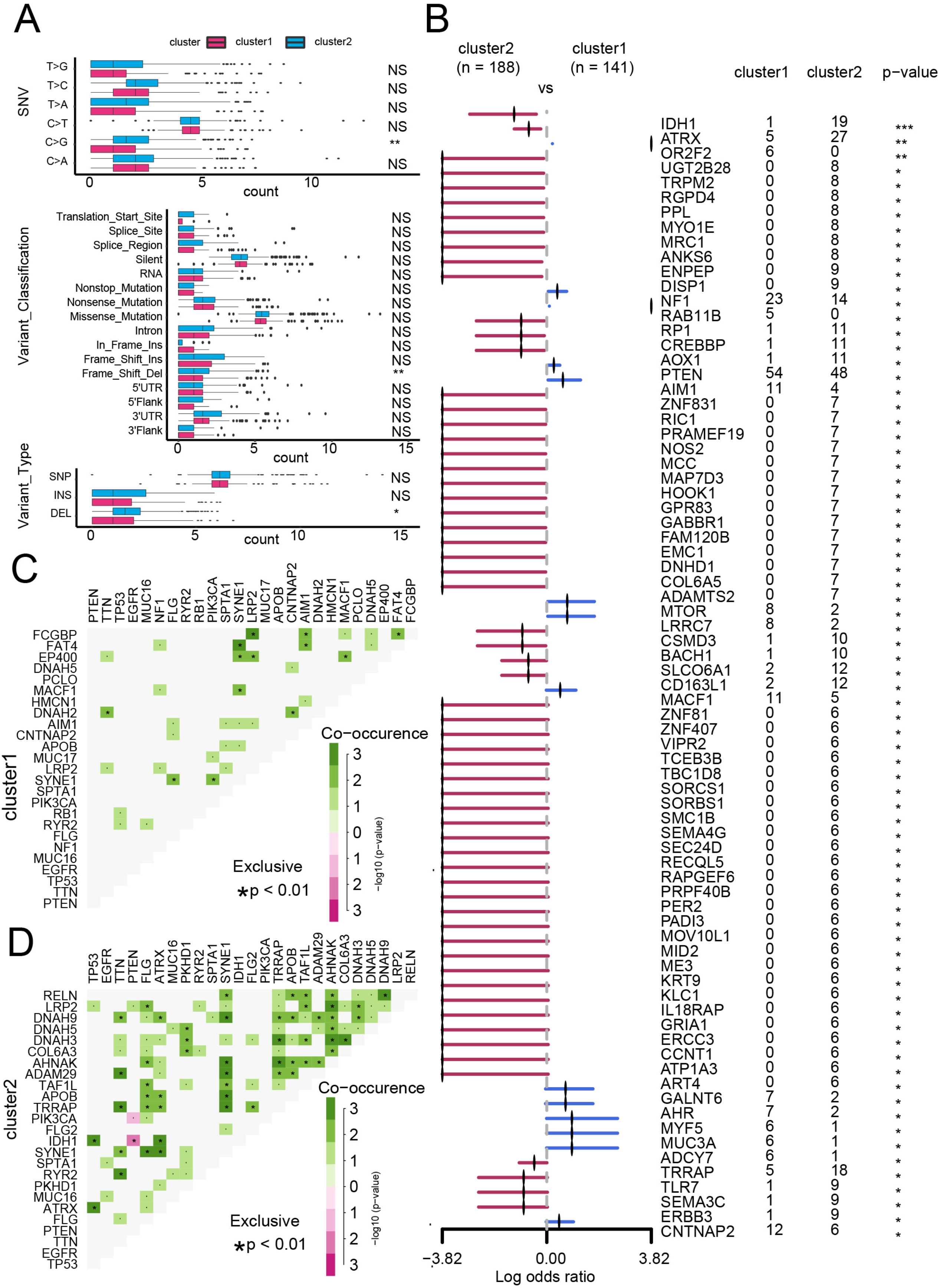
A
cluster
cluster1
cluster2
SNV
T>G NS
T>C NS
T>A NS
C>T NS
C>G **
C>A NS
0 5 10
count
Variant_Classification
Translation_Start_Site NS
Splice_Site NS
Splice_Region NS
Silent NS
RNA NS
Nonstop_Mutation NS
Nonsense_Mutation NS
Missense_Mutation NS
Intron NS
In_Frame_Ins NS
Frame_Shift_Ins NS
Frame_Shift_Del **
5'UTR NS
5'Flank NS
3'UTR NS
3'Flank NS
0 5 10 15
count
Variant_Type
SNP NS
INS NS
DEL *
0 5 10 15
count
B
cluster2 (n = 188)
vs
cluster1 (n = 141)
cluster1 cluster2 p-value
IDH1 1 19 ***
ATRX 5 27 **
OR2F2 6 0 **
UGT2B28 0 8 *
TRPM2 0 8 *
RGPD4 0 8 *
PPL 0 8 *
MYO1E 0 8 *
MRC1 0 8 *
ANKS6 0 8 *
ENPEP 0 9 *
DISP1 0 9 *
NF1 23 14 *
RAB11B 5 0 *
RP1 1 11 *
CREBBP 1 11 *
AOX1 1 11 *
PTEN 54 48 *
AIM1 11 4 *
ZNF831 0 7 *
RIC1 0 7 *
PRAMEF19 0 7 *
NOS2 0 7 *
MCC 0 7 *
MAP7D3 0 7 *
HOOK1 0 7 *
GPR83 0 7 *
GABBR1 0 7 *
FAM120B 0 7 *
EMC1 0 7 *
DNHD1 0 7 *
COL6A5 0 7 *
ADAMTS2 0 7 *
MTOR 8 2 *
LRRC7 8 2 *
CSMD3 1 10 *
BACH1 1 10 *
SLCO6A1 2 12 *
CD163L1 2 12 *
MACF1 11 5 *
ZNF81 0 6 *
ZNF407 0 6 *
VIPR2 0 6 *
TCEB3B 0 6 *
TBC1D8 0 6 *
SORCS1 0 6 *
SORBS1 0 6 *
SMC1B 0 6 *
SEMA4G 0 6 *
SEC24D 0 6 *
RECQL5 0 6 *
RAPGEF6 0 6 *
PRPF40B 0 6 *
PER2 0 6 *
PADI3 0 6 *
MOV10L1 0 6 *
MID2 0 6 *
ME3 0 6 *
KRT9 0 6 *
KLC1 0 6 *
IL18RAP 0 6 *
GRIA1 0 6 *
ERCC3 0 6 *
CCNT1 0 6 *
ATP1A3 0 6 *
ART4 0 6 *
GALNT6 7 2 *
AHR 7 2 *
MYF5 6 1 *
MUC3A 6 1 *
ADCY7 6 1 *
TRRAP 5 18 *
TLR7 1 9 *
SEMA3C 1 9 *
ERBB3 1 9 *
CNTNAP2 12 6 *
-3.82 0.00 3.82
Log odds ratio
C
PTEN TTN TP53 EGFR MUC16 NF1 FLG RYR2 RB1 PIK3CA SPTA1 SYNE1 LRP2 MUC17 APOB CNTNAP2 AIM1 DNAH2 HMCN1 MACF1 PCLO DNAH5 EP400 FAT4 FCGBP
FCGBP FAT4 EP400 DNAH5 PCLO MACF1 HMCN1 DNAH2 AIM1 CNTNAP2 APOB MUC17 LRP2 SYNE1 SPTA1 PIK3CA RB1 RYR2 FLG NF1 MUC16 EGFR TP53 TTN PTEN
cluster1
Co−occurence
3 2 1 0 1 2 3
−log10 (p−value)
Exclusive
*p < 0.01
D
TP53 EGFR TTN PTEN FLG ATRX MUC16 PKHD1 RYR2 SPTA1 SYNE1 IDH1 FLG2 PIK3CA TRRAP APOB TAF1L ADAM29 AHNAK COL6A3 DNAH3 DNAH5 DNAH9 LRP2 RELN
RELN LRP2 DNAH9 DNAH5 DNAH3 COL6A3 AHNAK ADAM29 TAF1L APOB TRRAP PIK3CA FLG2 IDH1 SYNE1 SPTA1 RYR2 PKHD1 MUC16 ATRX FLG PTEN TTN EGFR TP53
cluster2
Co−occurence
3 2 1 0 1 2 3
−log10 (p−value)
Exclusive
*p < 0.01

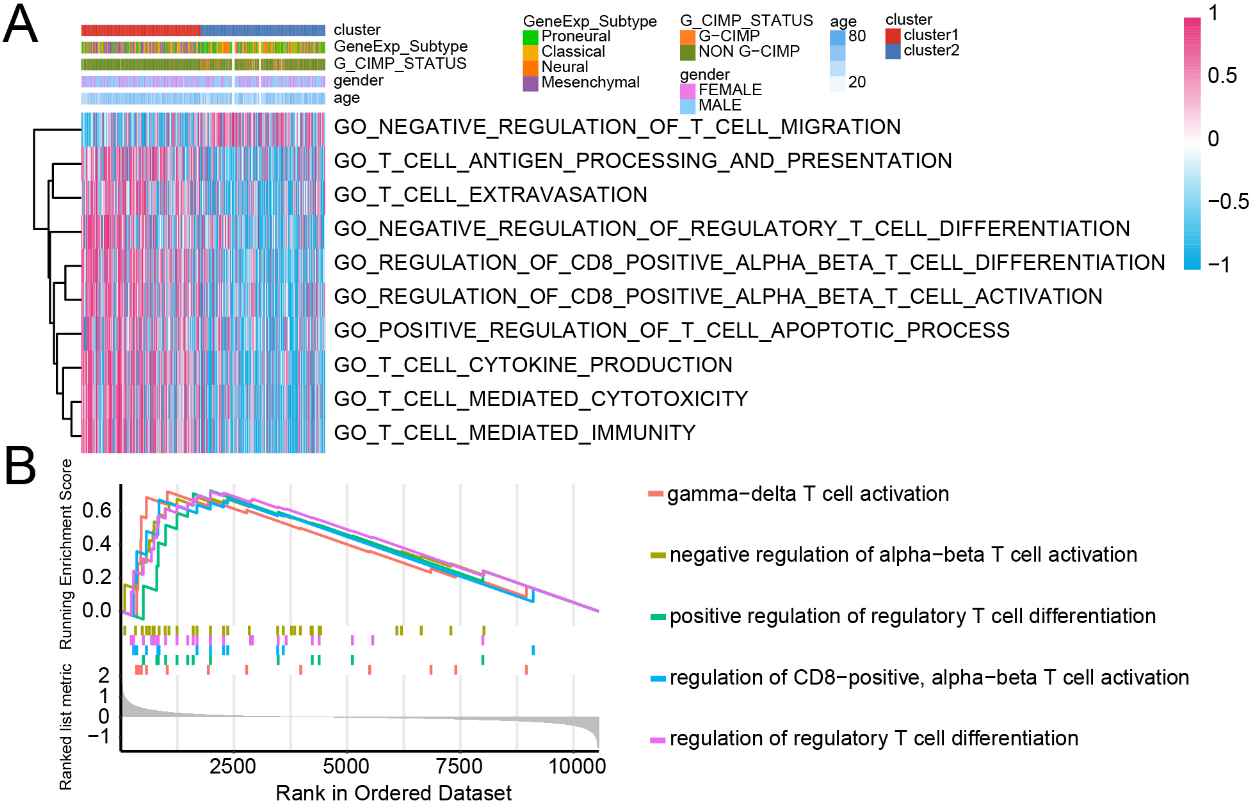

A
cluster
GeneExp_Subtype
G_CIMP_STATUS
gender
age
GeneExp_Subtype
Proneural
Classical
Neural
Mesenchymal
G_CIMP_STATUS
G−CIMP
NON G−CIMP
gender
FEMALE
MALE
age
80
20
cluster
cluster1
cluster2
1
0.5
0
−0.5
−1
GO_NEGATIVE_REGULATION_OF_T_CELL_MIGRATION
GO_T_CELL_ANTIGEN_PROCESSING_AND_PRESENTATION
GO_T_CELL_EXTRAVASATION
GO_NEGATIVE_REGULATION_OF_REGULATORY_T_CELL_DIFFERENTIATION
GO_REGULATION_OF_CD8_POSITIVE_ALPHA_BETA_T_CELL_DIFFERENTIATION
GO_REGULATION_OF_CD8_POSITIVE_ALPHA_BETA_T_CELL_ACTIVATION
GO_POSITIVE_REGULATION_OF_T_CELL_APOPTOTIC_PROCESS
GO_T_CELL_CYTOKINE_PRODUCTION
GO_T_CELL_MEDIATED_CYTOTOXICITY
GO_T_CELL_MEDIATED_IMMUNITY
B
Running Enrichment Score
0.6
0.4
0.2
0.0
Ranked list metric
2
1
0
−1
2500
5000
7500
10000
Rank in Ordered Dataset
gamma−delta T cell activation
negative regulation of alpha−beta T cell activation
positive regulation of regulatory T cell differentiation
regulation of CD8−positive, alpha−beta T cell activation
regulation of regulatory T cell differentiation

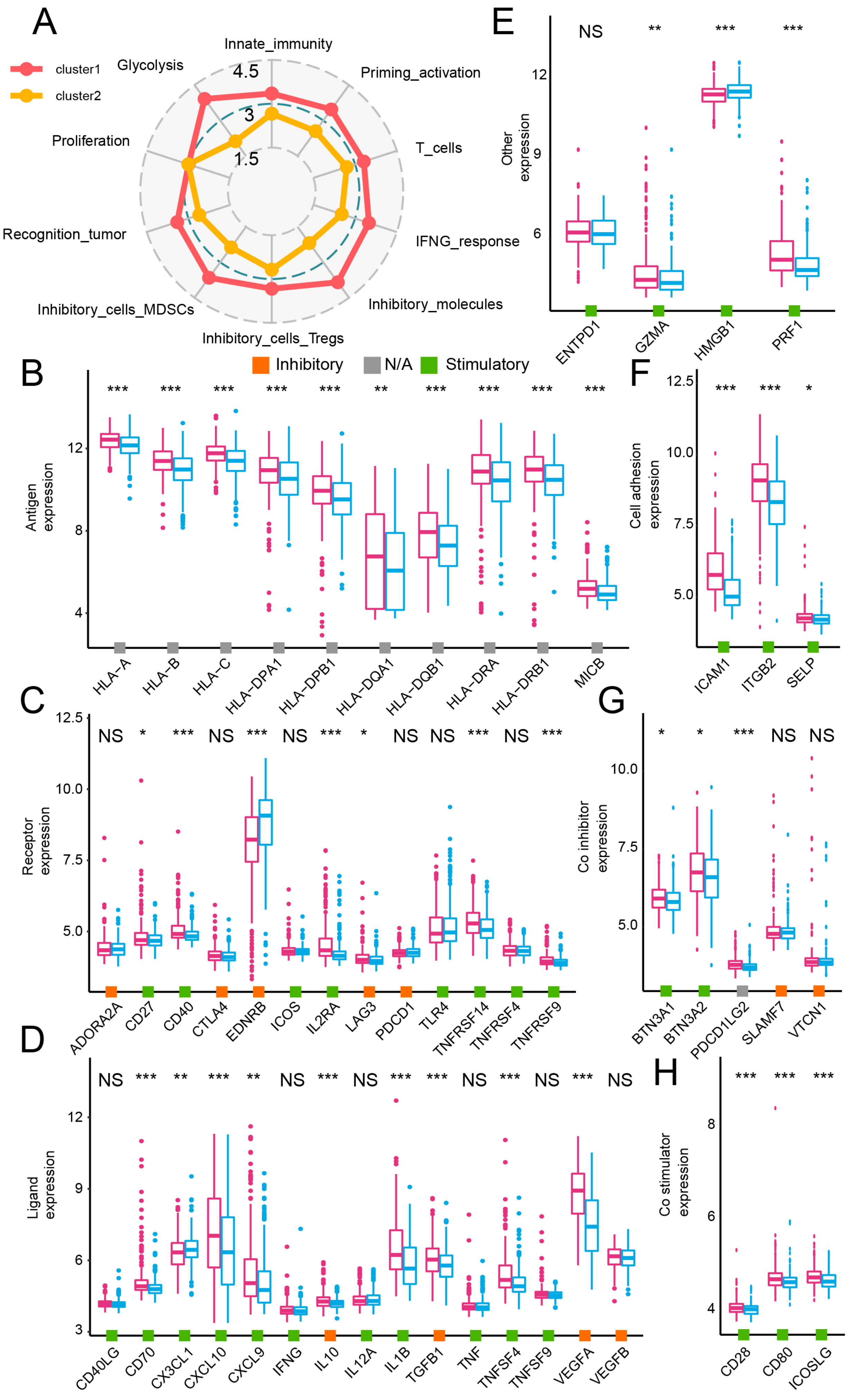

A
cluster1
cluster2
Innate_immunity
Priming_activation
T_cells
IFNG_response
Inhibitory_molecules
Inhibitory_cells_Tregs
Inhibitory_cells_MDSCs
Recognition_tumor
Proliferation
Glycolysis
4.5
3
1.5
E
Other expression
NS ** *** ***
ENTPD1
GZMA
HMGB1
PRF1
Inhibitory
N/A
Stimulatory
B
Antigen expression
HLA-A
HLA-B
HLA-C
HLA-DPA1
HLA-DPB1
HLA-DQA1
HLA-DQB1
HLA-DRA
HLA-DRB1
MICB
F
Cell adhesion expression
ICAM1
ITGB2
SELP
C
Receptor expression
ADORA2A
CD27
CD40
CTLA4
EDNRB
ICOS
IL2RA
LAG3
PDCD1
TLR4
TNFRSF14
TNFRSF4
TNFRSF9
G
Co inhibitor expression
BTN3A1
BTN3A2
PDCD1LG2
SLAMF7
VTCN1
D
Ligand expression
CD40LG
CD70
CX3CL1
CXCL10
CXCL9
IFNG
IL10
IL12A
IL1B
TGFB1
TNF
TNFSF4
TNFSF9
VEGFA
VEGFB
H
Co stimulator expression
CD28
CD80
ICOSLG

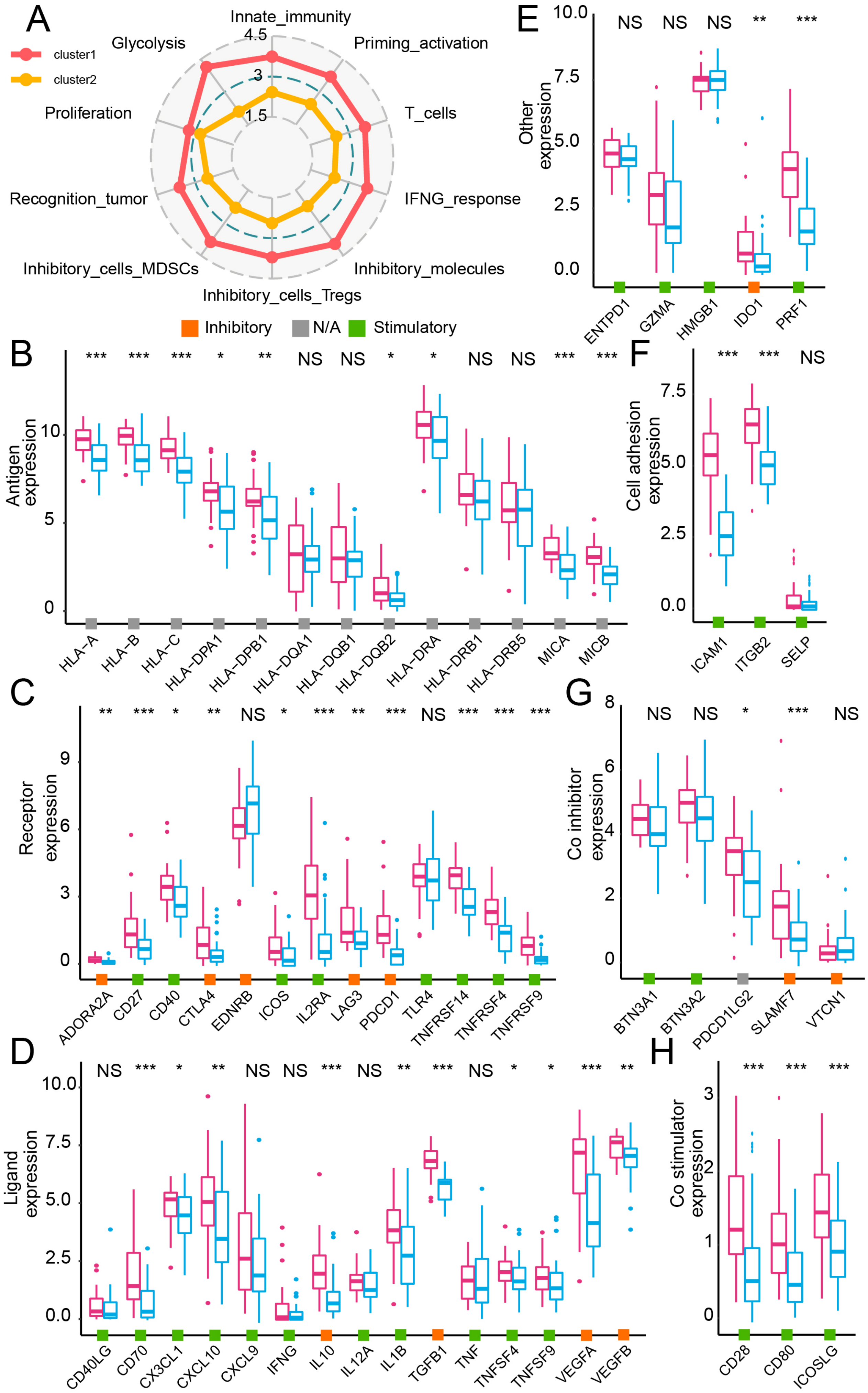
A
B
C
D
E
F
G
H
cluster1
cluster2
Innate_immunity
Priming_activation
T_cells
IFNG_response
Inhibitory_molecules
Inhibitory_cells_Tregs
Inhibitory_cells_MDSCs
Recognition_tumor
Proliferation
Glycolysis
4.5
3
1.5
Inhibitory
N/A
Stimulatory
Antigen expression
HLA-A
HLA-B
HLA-C
HLA-DPA1
HLA-DPB1
HLA-DQA1
HLA-DQB1
HLA-DQB2
HLA-DRA
HLA-DRB1
HLA-DRB5
MICA
MICB
Receptor expression
ADORA2A
CD27
CD40
CTLA4
EDNRB
ICOS
IL2RA
LAG3
PDCD1
TLR4
TNFRSF14
TNFRSF4
TNFRSF9
Ligand expression
CD40LG
CD70
CX3CL1
CXCL10
CXCL9
IFNG
IL10
IL12A
IL1B
TGFB1
TNF
TNFSF4
TNFSF9
VEGFA
VEGFB
Other expression
ENTPD1
GZMA
HMGB1
IDO1
PRF1
Cell adhesion expression
ICAM1
ITGB2
SELP
Co inhibitor expression
BTN3A1
BTN3A2
PDCD1LG2
SLAMF7
VTCN1
Co stimulator expression
CD28
CD80
ICOSLG

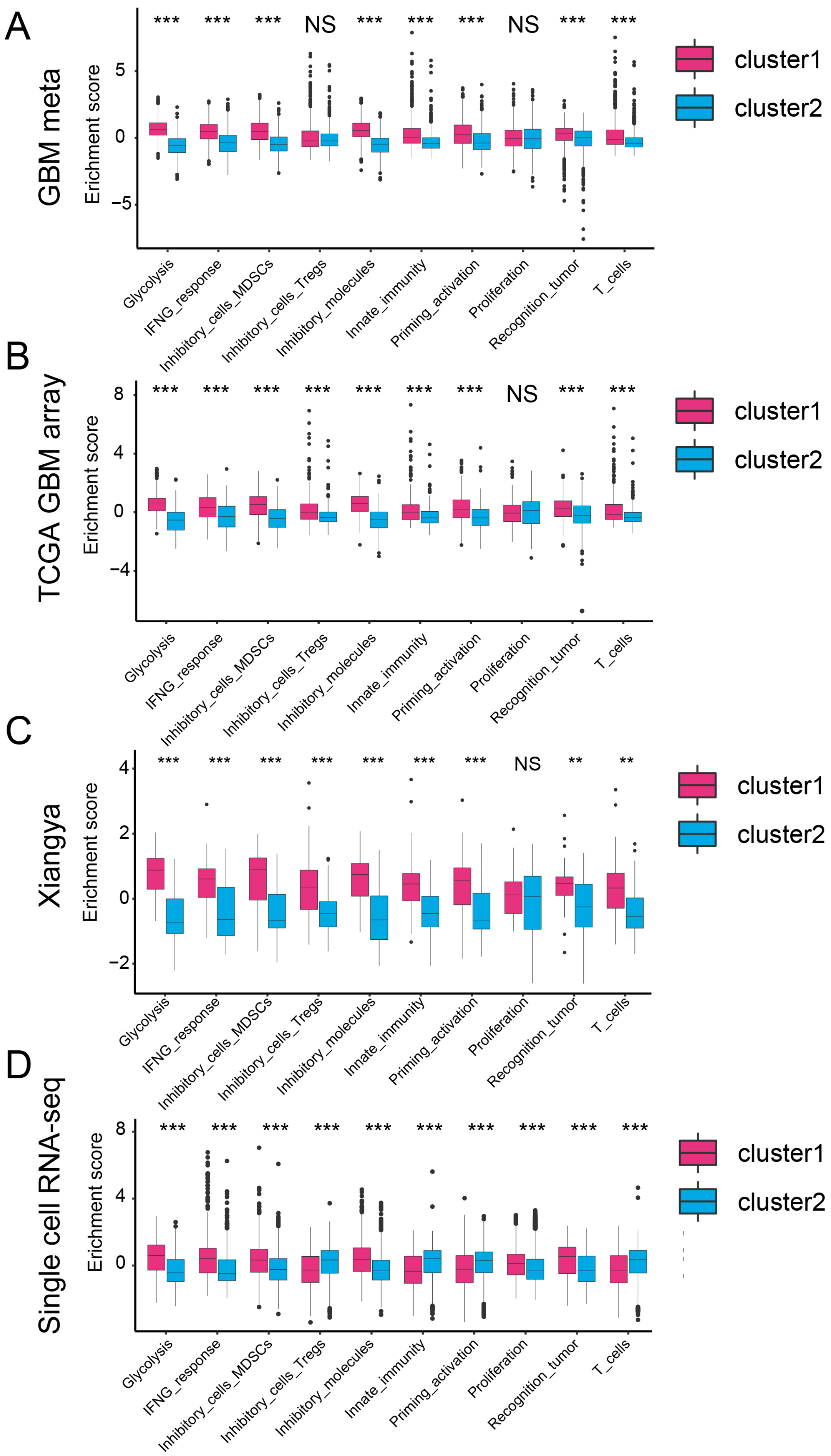
A
GBM meta
Erichment score
*** *** *** NS *** *** *** NS *** ***
cluster1
cluster2
Glycolysis
IFNG_response
Inhibitory_cells_MDSCs
Inhibitory_cells_Tregs
Inhibitory_molecules
Innate_immunity
Priming_activation
Proliferation
Recognition_tumor
T_cells
B
TCGA GBM array
Erichment score
*** *** *** *** *** *** *** NS *** ***
cluster1
cluster2
Glycolysis
IFNG_response
Inhibitory_cells_MDSCs
Inhibitory_cells_Tregs
Inhibitory_molecules
Innate_immunity
Priming_activation
Proliferation
Recognition_tumor
T_cells
C
Xiangya
Erichment score
*** *** *** *** *** *** *** NS ** **
cluster1
cluster2
Glycolysis
IFNG_response
Inhibitory_cells_MDSCs
Inhibitory_cells_Tregs
Inhibitory_molecules
Innate_immunity
Priming_activation
Proliferation
Recognition_tumor
T_cells
D
Single cell RNA-seq
Erichment score
*** *** *** *** *** *** *** *** *** ***
cluster1
cluster2
Glycolysis
IFNG_response
Inhibitory_cells_MDSCs
Inhibitory_cells_Tregs
Inhibitory_molecules
Innate_immunity
Priming_activation
Proliferation
Recognition_tumor
T_cells

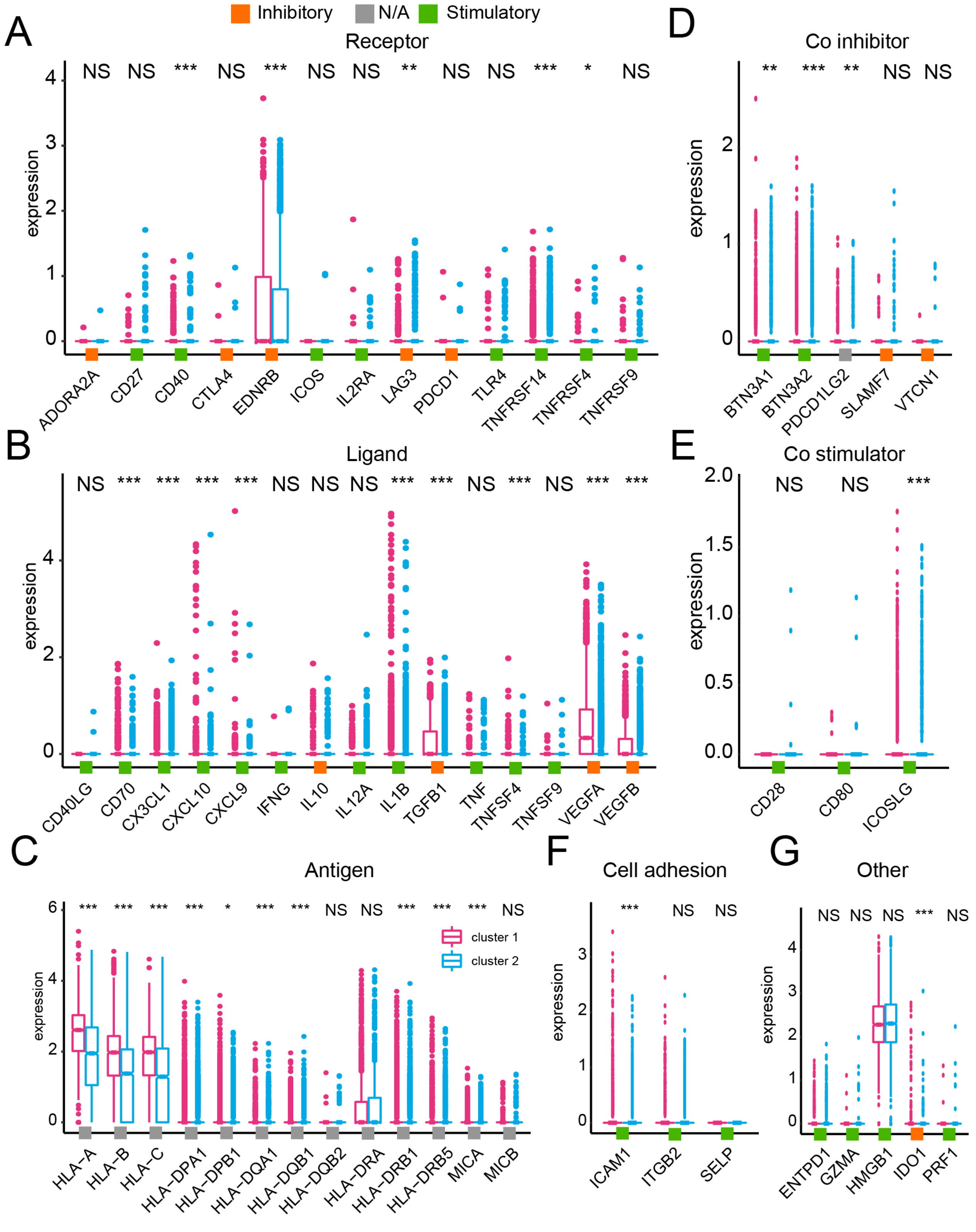

Inhibitory
N/A
Stimulatory
A
Receptor
NS NS *** NS *** NS NS ** NS NS *** * NS
expression
ADORA2A
CD27
CD40
CTLA4
EDNRB
ICOS
IL2RA
LAG3
PDCD1
TLR4
TNFRSF14
TNFRSF4
TNFRSF9
D
Co inhibitor
** *** ** NS NS
BTN3A1
BTN3A2
PDCD1LG2
SLAMF7
VTCN1
B
Ligand
NS *** *** *** *** NS NS NS *** *** NS *** NS *** ***
CD40LG
CD70
CX3CL1
CXCL10
CXCL9
IFNG
IL10
IL12A
IL1B
TGFB1
TNF
TNFSF4
TNFSF9
VEGFA
VEGFB
E
Co stimulator
NS NS ***
CD28
CD80
ICOSLG
C
Antigen
*** *** *** *** * *** *** NS NS *** *** *** NS
cluster 1
cluster 2
HLA-A
HLA-B
HLA-C
HLA-DPA1
HLA-DPB1
HLA-DQA1
HLA-DQB1
HLA-DQB2
HLA-DRA
HLA-DRB1
HLA-DRB5
MICA
MICB
F
Cell adhesion
*** NS NS
ICAM1
ITGB2
SELP
G
Other
NS NS NS *** NS
ENTPD1
GZMA
HMGB1
IDO1
PRF1

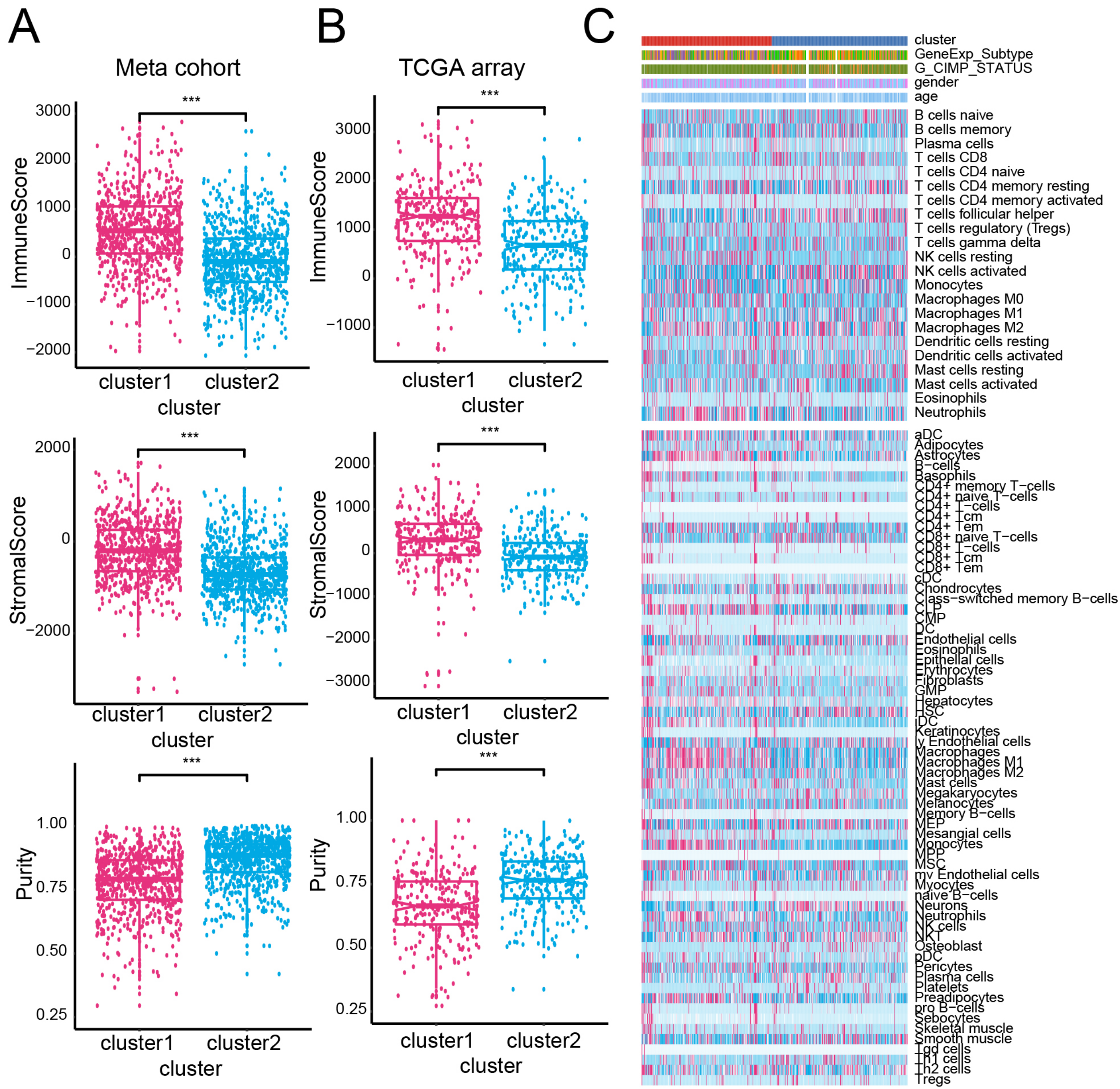
A
Meta cohort
ImmuneScore
***
cluster1
cluster2
cluster
StromalScore
Purity
B
TCGA array
C
cluster
GeneExp_Subtype
G_CIMP_STATUS
gender
age
B cells naive
B cells memory
Plasma cells
T cells CD8
T cells CD4 naive
T cells CD4 memory resting
T cells CD4 memory activated
T cells follicular helper
T cells regulatory (Tregs)
T cells gamma delta
NK cells resting
NK cells activated
Monocytes
Macrophages M0
Macrophages M1
Macrophages M2
Dendritic cells resting
Dendritic cells activated
Mast cells resting
Mast cells activated
Eosinophils
Neutrophils
aDC
Adipocytes
Astrocytes
B−cells
Basophils
CD4+ memory T−cells
CD4+ naive T−cells
CD4+ T−cells
CD4+ Tcm
CD4+ Tem
CD8+ naive T−cells
CD8+ T−cells
CD8+ Tcm
CD8+ Tem
cDC
Chondrocytes
Class−switched memory B−cells
CLP
CMP
DC
Endothelial cells
Eosinophils
Epithelial cells
Erythrocytes
Fibroblasts
GMP
Hepatocytes
HSC
iDC
Keratinocytes
ly Endothelial cells
Macrophages
Macrophages M1
Macrophages M2
Mast cells
Megakaryocytes
Melanocytes
Memory B−cells
MEP
Mesangial cells
Monocytes
MPP
MSC
mv Endothelial cells
Myocytes
naive B−cells
Neurons
Neutrophils
NK cells
NKT
Osteoblast
pDC
Pericytes
Plasma cells
Platelets
Preadipocytes
pro B−cells
Sebocytes
Skeletal muscle
Smooth muscle
Tgd cells
Th1 cells
Th2 cells
Tregs

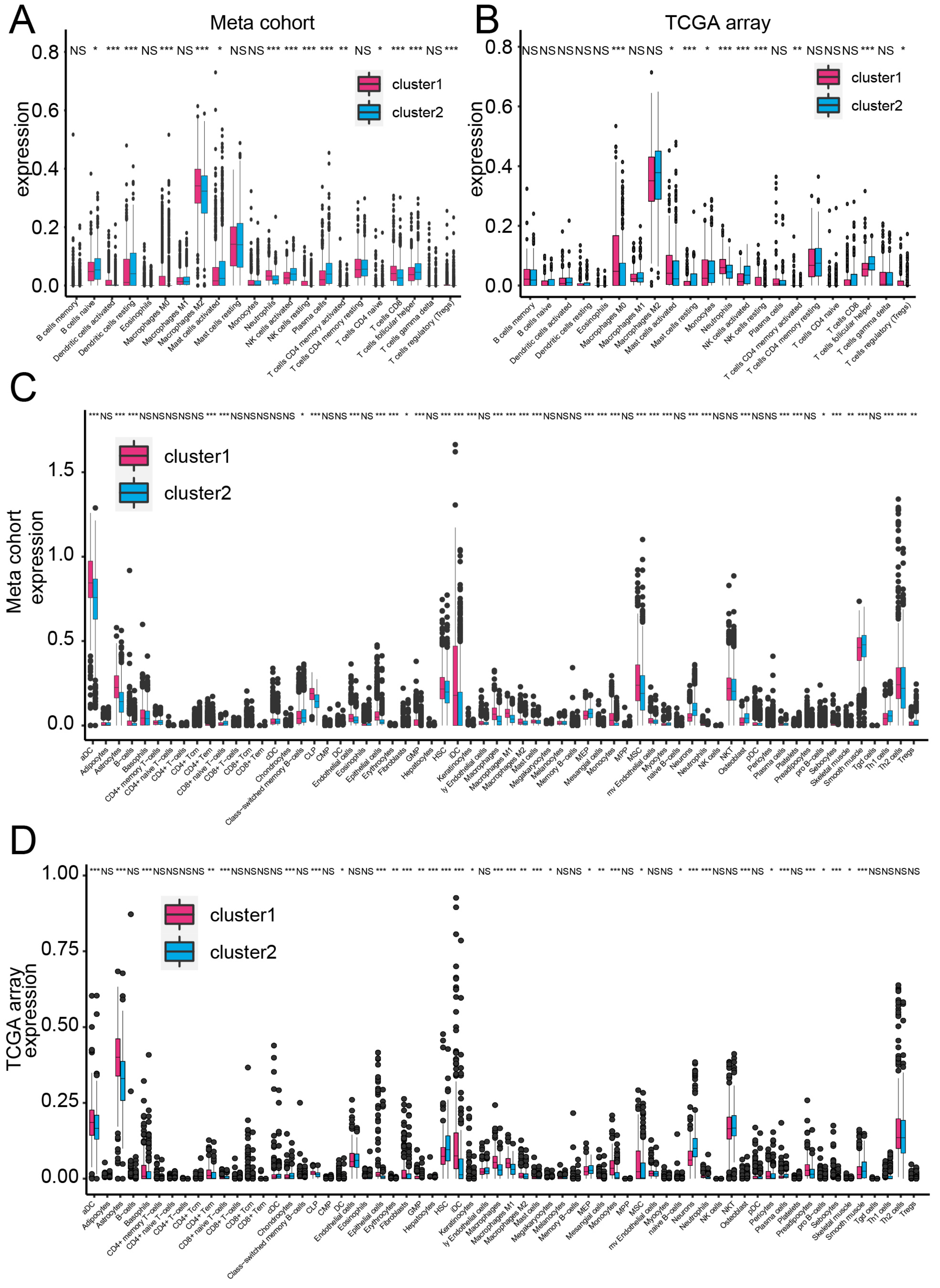

A
Meta cohort
expression
0.0
0.2
0.4
0.6
0.8
cluster1
cluster2
B cells memory
B cells naive
Dendritic cells activated
Dendritic cells resting
Eosinophils
Macrophages M0
Macrophages M1
Macrophages M2
Mast cells activated
Mast cells resting
Monocytes
Neutrophils
NK cells activated
NK cells resting
Plasma cells
T cells CD4 memory activated
T cells CD4 memory resting
T cells CD4 naive
T cells CD8
T cells follicular helper
T cells gamma delta
T cells regulatory (Tregs)
B
TCGA array
expression
0.0
0.2
0.4
0.6
0.8
cluster1
cluster2
B cells memory
B cells naive
Dendritic cells activated
Dendritic cells resting
Eosinophils
Macrophages M0
Macrophages M1
Macrophages M2
Mast cells activated
Mast cells resting
Monocytes
Neutrophils
NK cells activated
NK cells resting
Plasma cells
T cells CD4 memory activated
T cells CD4 memory resting
T cells CD4 naive
T cells CD8
T cells follicular helper
T cells gamma delta
T cells regulatory (Tregs)
C
Meta cohort expression
0.0
0.5
1.0
1.5
cluster1
cluster2
aDC
Adipocytes
Astrocytes
B-cells
Basophils
CD4+ memory T-cells
CD4+ naive T-cells
CD4+ T-cells
CD4+ Tcm
CD4+ Tem
CD8+ naive T-cells
CD8+ T-cells
CD8+ Tcm
CD8+ Tem
cDC
Chondrocytes
Class-switched memory B-cells
CLP
CMP
DC
Endothelial cells
Eosinophils
Epithelial cells
Erythrocytes
Fibroblasts
GMP
Hepatocytes
HSC
iDC
Keratinocytes
ly Endothelial cells
Macrophages
Macrophages M1
Macrophages M2
Mast cells
Megakaryocytes
Melanocytes
Memory B-cells
MEP
Mesangial cells
Monocytes
MPP
MSC
mv Endothelial cells
Myocytes
naive B-cells
Neurons
Neutrophils
NK cells
NKT
Osteoblast
pDC
Pericytes
Plasma cells
Platelets
Preadipocytes
pro B-cells
Sebocytes
Skeletal muscle
Smooth muscle
Tgd cells
Th1 cells
Th2 cells
Tregs
D
TCGA array expression
0.00
0.25
0.50
0.75
1.00
cluster1
cluster2
aDC
Adipocytes
Astrocytes
B-cells
Basophils
CD4+ memory T-cells
CD4+ naive T-cells
CD4+ T-cells
CD4+ Tcm
CD4+ Tem
CD8+ naive T-cells
CD8+ T-cells
CD8+ Tcm
CD8+ Tem
cDC
Chondrocytes
Class-switched memory B-cells
CLP
CMP
DC
Endothelial cells
Eosinophils
Epithelial cells
Erythrocytes
Fibroblasts
GMP
Hepatocytes
HSC
iDC
Keratinocytes
ly Endothelial cells
Macrophages
Macrophages M1
Macrophages M2
Mast cells
Megakaryocytes
Melanocytes
Memory B-cells
MEP
Mesangial cells
Monocytes
MPP
MSC
mv Endothelial cells
Myocytes
naive B-cells
Neurons
Neutrophils
NK cells
NKT
Osteoblast
pDC
Pericytes
Plasma cells
Platelets
Preadipocytes
pro B-cells
Sebocytes
Skeletal muscle
Smooth muscle
Tgd cells
Th1 cells
Th2 cells
Tregs

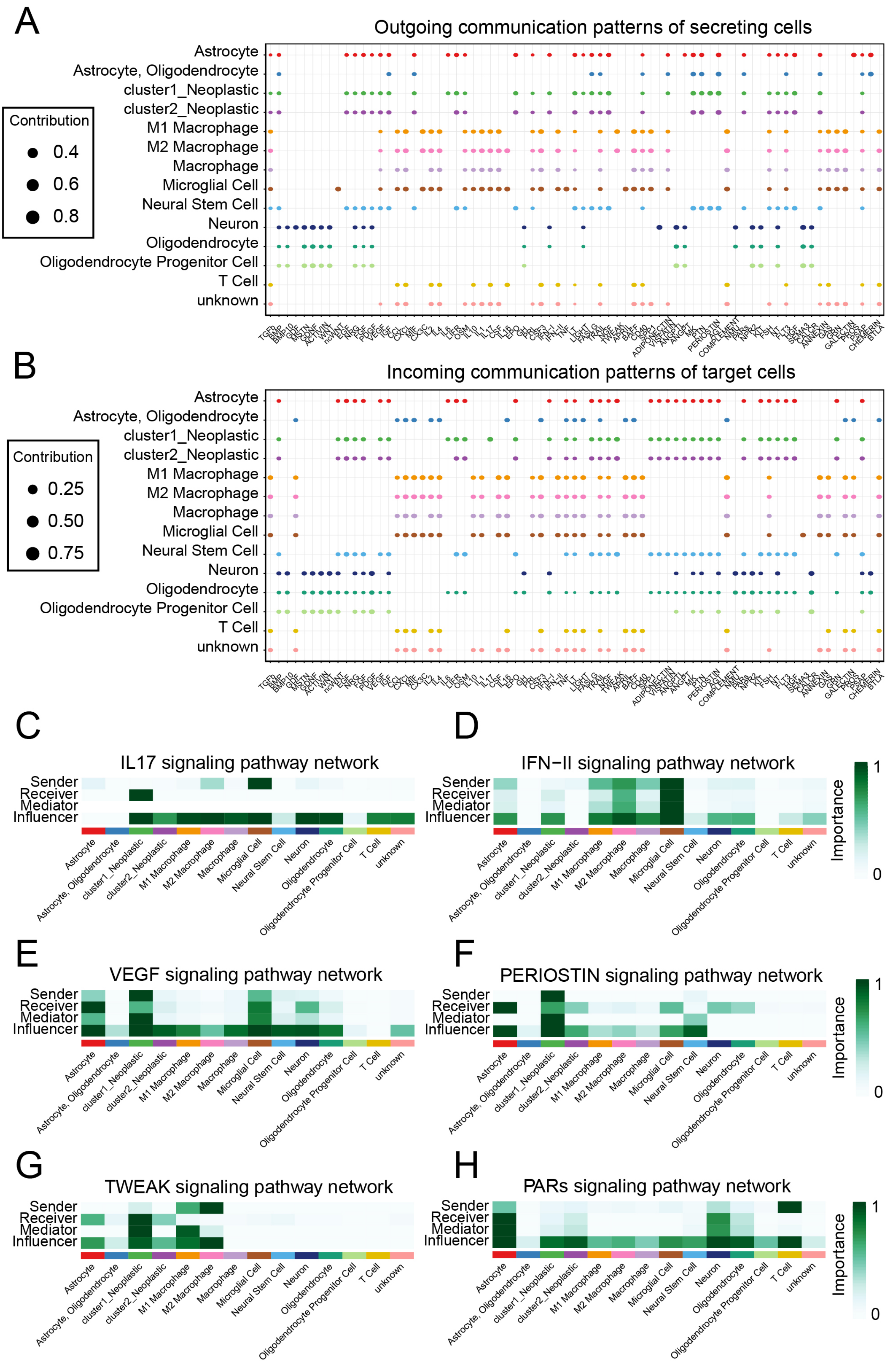
A
Outgoing communication patterns of secreting cells
Contribution
0.4
0.6
0.8
Astrocyte
Astrocyte, Oligodendrocyte
cluster1_Neoplastic
cluster2_Neoplastic
M1 Macrophage
M2 Macrophage
Macrophage
Microglial Cell
Neural Stem Cell
Neuron
Oligodendrocyte
Oligodendrocyte Progenitor Cell
T Cell
unknown
TGFb BMP BMP10 GDF MSTN GDNF ACTIVIN WNT ncWNT EGF NRG FGF PDGF VEGF IGF CCL CXCL MIF CX3C IL2 IL4 IL6 LIFR OSM IL10 IL1 IL17 CSF IL16 EPO GH PRL CSF3 IFN-I IFN-II TNF LT LIGHT FASLG TRAIL NGF TWEAK APRIL BAFF CD40 SPP1 ADIPONECTIN VISFATIN ANGPTL ANGPT MK PTN PERIOSTIN AGT COMPLEMENT NMU PARs NPR2 KIT FSH NT FLT3 HGF SEMA3 CALCR ANNEXIN GAS GRN GALECTIN PROS PSAP CHEMERIN BTLA
B
Incoming communication patterns of target cells
Contribution
0.25
0.50
0.75
C
IL17 signaling pathway network
Sender
Receiver
Mediator
Influencer
D
IFN−II signaling pathway network
Importance
1
0
E
VEGF signaling pathway network
F
PERIOSTIN signaling pathway network
G
TWEAK signaling pathway network
H
PARs signaling pathway network

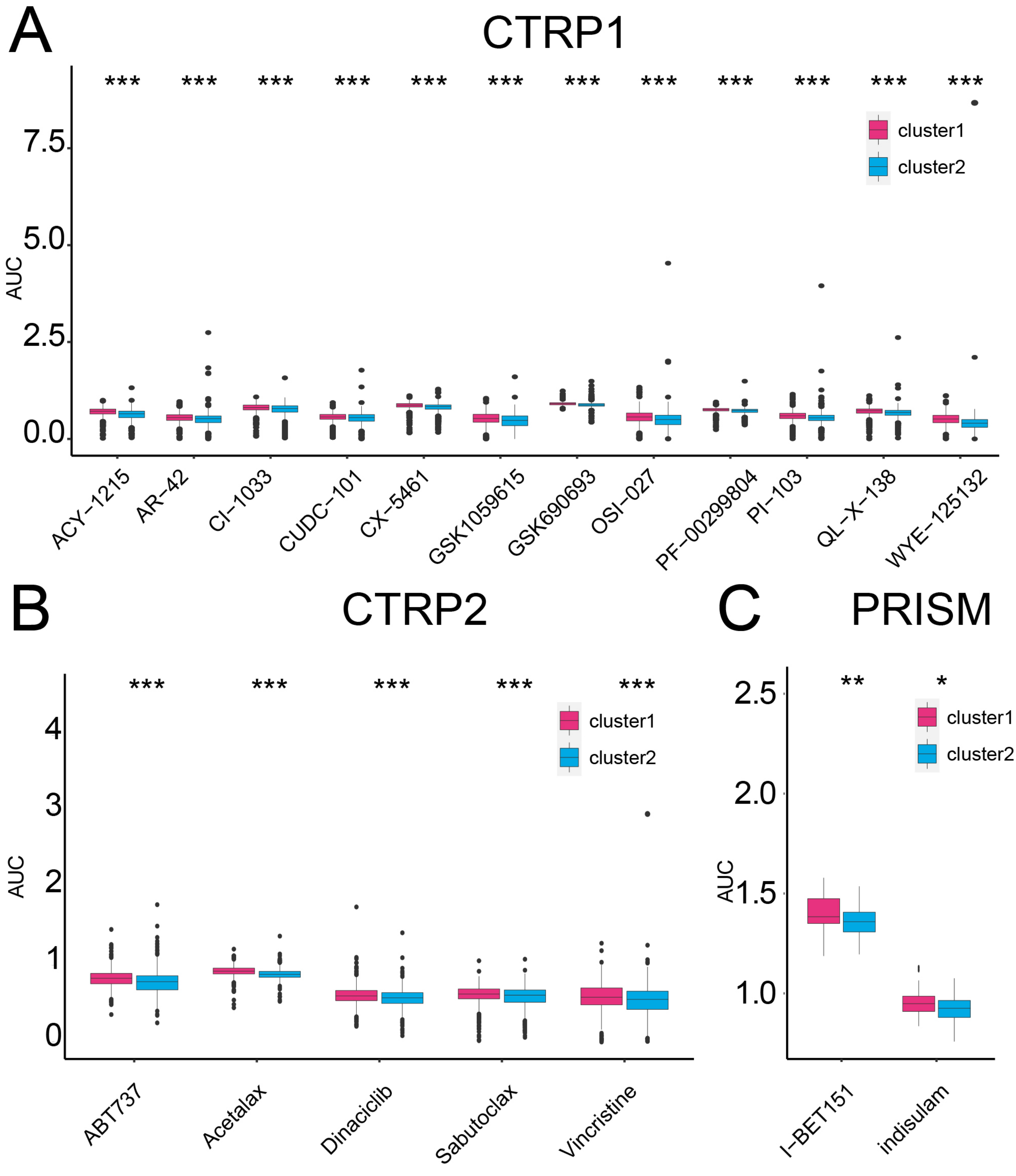
A
CTRP1
AUC
cluster1
cluster2
ACY−1215
AR−42
CI−1033
CUDC−101
CX−5461
GSK1059615
GSK690693
OSI−027
PF−00299804
PI−103
QL−X−138
WYE−125132
B
CTRP2
AUC
ABT737
Acetalax
Dinaciclib
Sabutoclax
Vincristine
C
PRISM
I−BET151
indisulam
